# Water-Soluble Trityl Radicals for Fluorescence Imaging

**DOI:** 10.3390/molecules29050995

**Published:** 2024-02-25

**Authors:** Mona E. Arnold, Larissa Schoeneburg, Markus Lamla, Alexander J. C. Kuehne

**Affiliations:** Institute of Macromolecular and Organic Chemistry, Ulm University, Albert-Einstein-Allee 11, 89081 Ulm, Germany

**Keywords:** triaryl methyl radicals, light-emitting radicals, cell culture, charge transfer states, photoluminescence quantum yield

## Abstract

Stable tris(trichlorophenyl)methyl radicals have gained interest as all-organic bioimaging agents combining fluorescent and paramagnetic properties. However, cellular uptake has so far only been reported for nanoparticles, because molecular hydrophobic trityl radicals are not soluble in aqueous media. Here, we report the synthesis and characterization of new water-soluble tris(trichlorophenyl)methyl radical derivatives exhibiting red doublet emission. Solubility in water is achieved through functionalization with oligoethylene glycol (OEG) chains. The emission behavior of OEG functionalized trityl radicals is studied in polar environments. Donor-functionalization with carbazole evokes a charge-transfer excited state that is efficiently quenched in polar solvents. In contrast, click-reaction mediated attachment of OEG-azide and trityl acetylene furnishes a triazole functionalized radical with locally excited states and emission in water. Confocal fluorescence microscopy proves successful uptake of the material by macrophages in cell culture, showing the potential of our water soluble trityl radical for fluorescence bioimaging.

## 1. Introduction

The stable tris(2,4,6-trichlorophenyl)methyl radical (TTM) and its derivatives represent a class of fluorescent open-shell molecules with emission in the orange to near-infrared spectrum [1,2,3]. Because of their doublet ground and excited state character, these molecules represent interesting candidates to overcome the problem of spin statistics in conventional closed-shell emitters, which is a limiting factor for the efficiency in organic light-emitting devices (OLEDs) [2,4,5,6]. Finland-radicals, a subclass of non-fluorescent trityl radicals that can be rendered water soluble, have been employed as magnetic probes in biomedical EPR imaging. These radical spin labels exhibit low toxicity and a narrow single-line resonance, enabling high contrast and superior image quality [7]. In contrast, TTM is paramagnetic as well as fluorescent, and therefore represents a promising candidate for dual-mode bioimaging [8]. TTM radicals that are functionalized with charged groups are water soluble; however, these radicals do not exhibit fluorescence [9,10]. Because of this quenched fluorescence or the limited solubility in aqueous media, neutral TTM radical-based imaging probes have been limited to nanoparticles that were explored in vitro and in vivo for their optical imaging quality [11,12,13]. However, the formulation of small molecular TTM radicals into an inert matrix or the polymerization of the radicals into particles requires large amounts of inert and non-magnetic carrier material. As a result, the nanoparticles are amorphous, and any beneficial coupling between the radical species will only take place, if at all, at very low temperatures. To be able to target certain compartments in a cell and to make use of the unpaired radical electron as spin labels or for magnetic field sensing, molecularly dissolved radicals are required.

Here, we report the synthesis of unprecedented TTM derivatives that are functionalized with oligoethylene glycol (OEG) chains, rendering the molecules soluble in polar solvents and aqueous media. In contrast to previous water-soluble TTM radicals, our derivatives exhibit red emission in water with properties similar to those of TTM in non-polar solvents. We see that radicals with charge transfer (CT) excited state, which are performant emitters in non-polar solvents, are inferior in polar and aqueous media. In contrast, radicals with a locally excited (LE) state exhibit luminescence in non-polar as well as in polar and aqueous media.

## 2. Results and Discussion

### 2.1. Synthesis

To address the challenge of equipping TTM radicals with water solubility and molecular dissolution, while keeping the molecule charge neutral, we functionalize TTM with OEG chains. OEGylation, as well as functionalization with polyethylene glycol (PEGylation), are typical pathways in biomedical materials science for endowing molecules with water solubility.

**Figure 1:**
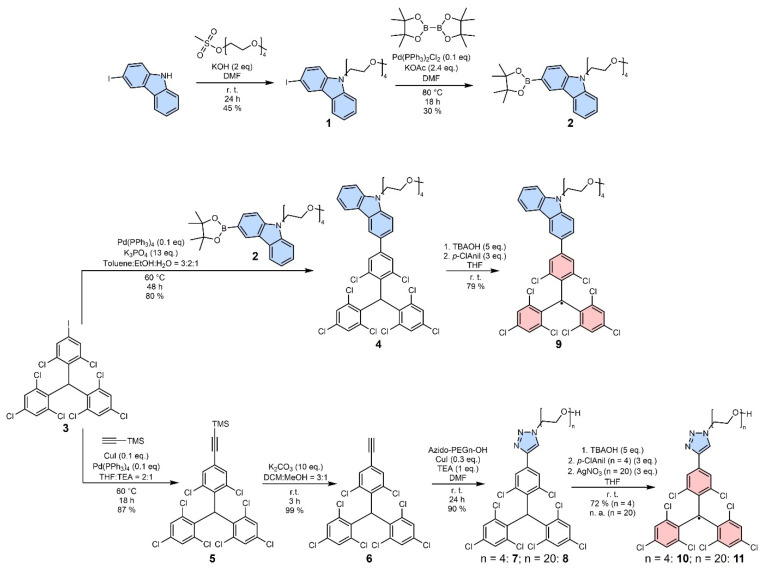
Synthesis of new OEG-functionalized TTM derivatives. The HTTM precursor carrying the N-OEGylated carbazole (**4**) is synthesized via Suzuki coupling of HTTM-I (**3**) and the boronic ester **2** [16]. HTTM functionalized with OEG chains of different length (**7** & **8**) with triazole as a linker is obtained from alkyne-azide Click-reaction with alkyne-HTTM (**6**). The latter is obtained from deprotection of **5** which is synthesized from **3** by Sonogashira-coupling using TMS-acetylene. The radicals are obtained from their respective precursors by deprotonation followed by mild oxidation.

TTM-derived radical emitters with reported high photoluminescence efficiency typically possess CT excited states [3,14,15]. However, it is known that such polar excited states are prone to fluorescence quenching in polar solvents, as a polar medium stabilizes the CT state. Therefore, we chose to test TTM derivatives that will exhibit CT and LE states. Carbazole (Cz) is a well-established donor substituent for TTM, typically delivering a CT state and high photoluminescence quantum yields (*ϕ*) [3]. To improve the solubility of this TTM-Cz radical, we *N*-functionalize the Cz with an OEG chain (see Figure 1). Moreover, to compare the photoluminescence character of such a CT emitter to an LE emitter in a polar environment, we employ an electron-deficient triazole linker for OEGylation. Since the emission is expected to not significantly depend on the length of the OEG, we start with a tetraethylene glycol (*n* = 4), despite the reduced capability to increase the solubility of our non-polar TTMs in water.

OEGylated carbazole **1** is obtained from 3-iodocarbazole, and borylation provides the building block **2** (see Figure 1). The OEGylation of both TTM-derivatives—with Cz or triazole—is achieved by starting from the iodinated HTTM **3** (HTTM-I, see Figure 1) [16]. HTTM-I reacts readily with the previously *N*-OEGylated carbazole **2** in a Suzuki cross-coupling reaction to give the donor-functionalized HTTM **4** in good yields and at mild reaction conditions. Alternatively, **3** can also be functionalized efficiently with an alkyne via Sonogashira cross-coupling to give Compound **5**. After cleavage of the TMS protecting group, the resulting HTTM-alkyne **6** is subjected to a copper-catalyzed alkyne-azide Click-reaction, to give the respective triazole-linked radical precursors **7** and **8** in excellent yield.

The compounds **4** and **7** are converted to the respective radicals **9** and **10** by deprotonation, followed by mild oxidation with *p*-chloranil of the resulting anions [17]. The open-shell nature of the resulting molecules is confirmed by EPR spectroscopy. The single signal indicates that the unpaired electron is mainly located at the methine carbon, as previously observed and described for related compounds (see Figure S2) [12,14,16,18].

### 2.2. Optical Characterization of Radicals Carrying OEG Chains in Organic Solvents

**Figure 2:**
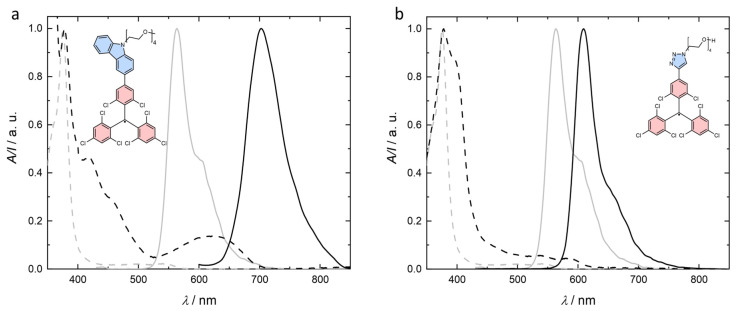
Absorption (dashed black) and emission (solid black) spectra of the radicals (at 0.1 ± 0.01 mM) measured in toluene solutions (10^−4^ M): (**a**) TTM-Cz *N*-functionalized with a short *n* = 4 OEG chains (**9**), (**b**) TTM-triazole with an *n* = 4 OEG chain (**10**). Absorption (dashed grey) and emission (solid grey) spectra for toluene solution of TTM are shown for comparison.

Both OEGylated TTM radicals **9** and **10** show weak absorption in the visible and strong absorption bands in the UV spectrum, as is typical for TTM and its derivatives (see Figure 2) [3,12,14,15,16,18]. The absorption in the visible region appears red-shifted with respect to TTM. For carbazole-functionalized TTM derivatives, this bathochromic shift in the absorption is related to a charge-transfer from the carbazole to the TTM moiety [19]. The low-energy absorption behavior of **9** is comparable to TTM carrying *N*-phenyl carbazole, indicating that *N*-PEGylation does not significantly affect the electronic properties of the donor substituent [20]. The low energy absorption of **10** appears to be hypsochromically shifted with respect to **9**. The electron deficient triazole attached to TTM will not act as an electron donor to TTM, and therefore the tendency for a CT excitation will be reduced. Still, the absorption is slightly red-shifted (*λ*_max_ = 582 nm) compared to unfunctionalized TTM (*λ*_max_ = 540 nm) (see Figure 2b) [17]. Despite triazole not acting as an electron-donor, the extension of the aromatic system into the triazole unit might be responsible for lowering of the absorption energy.

**Figure 3:**
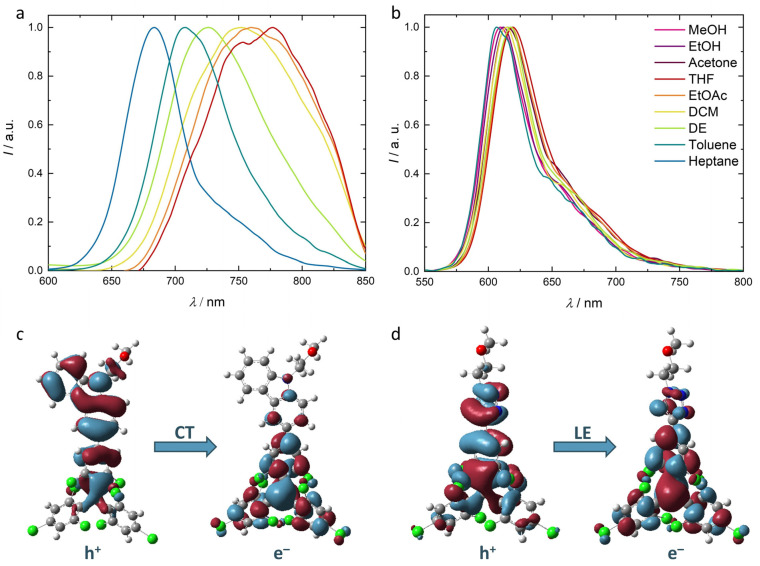
Emission spectra for solutions of radicals **9** (**a**) and **10** (**b**) in solvents of different polarity (0.1 ± 0.02 mM). While the emission maxima of **9** is red-shifted, with increasing solvent polarity, the emission of **10** is insensitive to the polarity of the environment. Natural transition orbitals (NTOs) for the D_0_ → D_1_ transition in **9** (**c**) and **10** (**d**) in their ground state geometry, calculated on the PBE0-GD3(BJ)/6-311++G(d,p), SCRF (SMD, water), level of theory.

To check the suitability of our new OEG substituted TTM derivatives as radical emitters in water, we record the photoluminescence spectra in different solvents of increasing polarity (see Figure 3a,b). For **9**, the emission maximum is clearly shifting with increasing solvent polarity, as is typical for CT excited states. Such charge–transfer upon excitation can be observed from the dependence of the Stokes shift on the orientation polarizability of the solvent in a Lippert–Mataga plot (see Figure S5). In addition, the spectra are vibrationally broadened, indicating strong interaction with the solvent molecules. For radical **9**, we observe a drop in the photoluminescence quantum yield from *ϕ* = 29% in heptane and 20% in diethyl ether to 4% in ethyl acetate. Quenching of the photoluminescence becomes even more efficient for highly polar solvents, such as acetone, ethanol, or methanol. We do not observe fluorescence of radical **9** for solutions of even higher solvent polarity like water. In contrast, the emission spectrum of **10** is hardly affected by the solvent polarity, which further supports our hypothesis from above that the excited state of radical **10** represents an LE state. The variation in the emission maxima in the different solvents lies within a range of 10 nm, while no clear trend is observable. This is in agreement with the observation from a Lippert–Mataga plot, also indicating the LE state (see Figure S5). Unfortunately, the solubility of **10** in water is too low in terms of studying its electronic properties in aqueous media. The value of *ϕ* is constantly low at 2% in solvents of different polarity, and also insensitive to the environment. While donor-substituted derivatives reach *ϕ* values close to unity, such molecules typically undergo CT excitation, such as observed for **9** [1,21]. However, for the application in polar environments, less efficient LE emissions, such as from **10,** appear more appropriate.

### 2.3. Quantum Chemical Calculations

To get further insight into the different emission behaviors of **9** and **10,** and support our hypotheses of CT and LE excited states, we employ density functional theory (DFT) and time-dependent density functional theory (TD-DFT) calculations on our radicals. As the length of the OEG-chain is expected to have a minor effect on the electronic properties of the excited state, it is shortened to a methoxy ethane group to reduce computation time. The molecules are optimized in their ground state geometry on the PBE0-GD3(BJ)/6-311++G(d,p) level of theory. The aqueous environment is simulated using the SCRF-SMD method. The natural transition orbitals (NTOs) for the D_1_ excitation in the ground state geometry for the radicals are shown in Figure 3c,d.

For most TTM derivatives, the D_1_ excitation is related to a HDMO-SOMO transition, while excitation of the unpaired electron appears at much higher energy [4,22,23]. Additionally, **9** shows a CT D_1_ excitation with a clear charge migration from the carbazole-donor to the TTM-acceptor moiety. Such behavior has been theoretically described for structurally related TTM derivatives connected to the 3-position of carbazole (see Figure 3c,d) [20,24]. As expected, there is no involvement of the aliphatic OEG substituent in the excitation, substantiating our omission of this unit for the calculations. The charge separation upon excitation explains the strong interaction of the molecule in the D_1_ state with its solvent environment. As the experimental observation of fluorescence quenching indicates, such polar excited states are unfavorable for emission in polar environments like water. The well-established concept of donor-functionalization for improved optical properties of TTM obviously works only for an environment of relatively low polarity [1,3]. In contrast, radical **10** shows a LE D_1_ excitation. While the NTO of the electron (e^−^) in **10** resembles that of radical **9**, the NTO of the hole (h^+^) is more strongly spread across the TTM moiety in **10** rather than in **9** (see Figure 3c,d). In addition, both hole and electron NTOs occupy the triazole substituent corroborating our hypothesis that the red-shifted absorption and emission spectra in radical **10** could originate from an extended π-conjugated system, with respect to unfunctionalized TTM. Such a π-extension of the conjugated system is supported by a planar configuration of the triazole and the attached phenyl ring of the TTM moiety, as is observed in the DFT optimized geometry of radical **10** (see Figure S4).

### 2.4. Water-Soluble TTM

To render the TTM-triazole-OEG radical water soluble, we increase the OEG chain length from *n* = 4 to *n* = 20 (see Figure 1). For the formation of the respective radical **11**, we use AgNO_3_ as an oxidizing agent instead of *p*-chloranil. This exchange simplifies the purification of the product by aqueous extraction rather than requiring column chromatography, which becomes increasingly tedious with increasing OEG chain lengths. In contrast to **10**, with an OEG chain length of *n* = 4, radical **11** with an OEG degree of polymerization of *n* = 20 is very well soluble in the aqueous medium. The solubility of **11** in water is determined to be at least 10 mg∙mL^−1^, which is far above the concentrations commonly used for fluorescent markers and tags in cell culture experiments.

**Figure 4:**
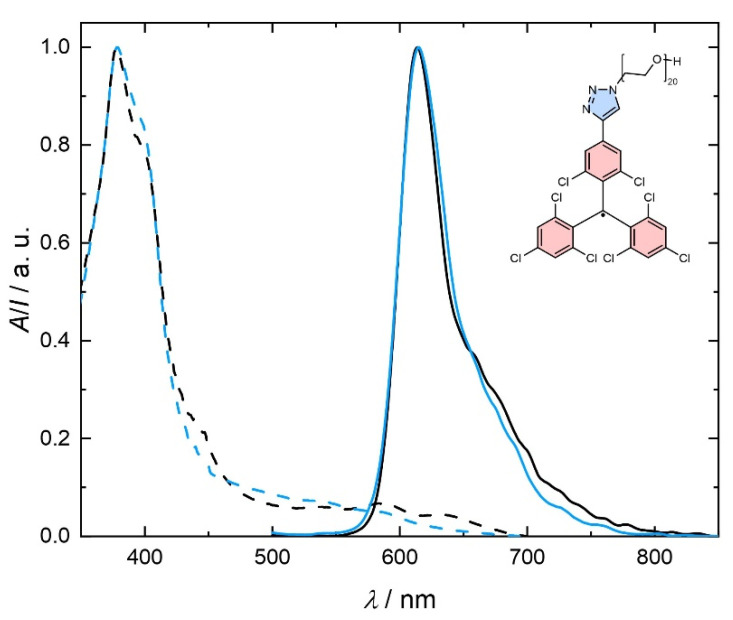
Absorption (dashed) and emission (solid) spectra for toluene (black) and water (blue) solutions of **11** (at 0.05 ± 0.01 mM).

Equipped with our insight gained from the study of **9** and **10** in polar solvents and the quantum chemical contemplations, we study compound **11** in aqueous solution and cell-culture experiments. Both, absorption and emission spectra for **11** in water resemble those of **10** with the much shorter OEG chain (see Figure 4). This observation confirms the minor impact of the length of the OEG chain on the electronic properties of the radicals, which are mainly governed by the aromatic units. Therefore, it is not surprising that the emission spectrum of **11** is also not greatly affected by solvent polarity, as expected for an emission originating from an LE state. Since many amphiphilic molecules form micelles in aqueous media, which could affect the optical properties, we perform dynamic light scattering (DLS) experiments with the non-emissive closed-shell precursor **8**. The latter is chosen to avoid interference of the fluorescent radical with the DLS laser wavelength (632.8 nm). In a concentration of 1 mM of **8** in water, we do not detect any aggregates. This concentration is well above the concentrations used for spectroscopy and cell culture experiments. Therefore, we conclude that the absorption and emission profiles correspond to individually dissolved molecules. The photoluminescence quantum yield of **11** in both toluene and water is *ϕ* = 2%. This invariance in performance in solvents of different polarity further substantiates our hypothesis of an LE state. These observations lead us to conclude that light emitting radicals for aqueous media should be designed to have an LE state, while CT excited states should be avoided. While *ϕ* = 2% does not seem to be high, this quantum yield is in the range of those reported for TTM without further functionalization in non-polar solvents [1,14,15,16]. As discussed above, much higher *ϕ* are possible in donor-functionalized TTM radicals; however, these cannot be transferred into polar media without photoluminescence quenching and loss of performance.

**Figure 5:**
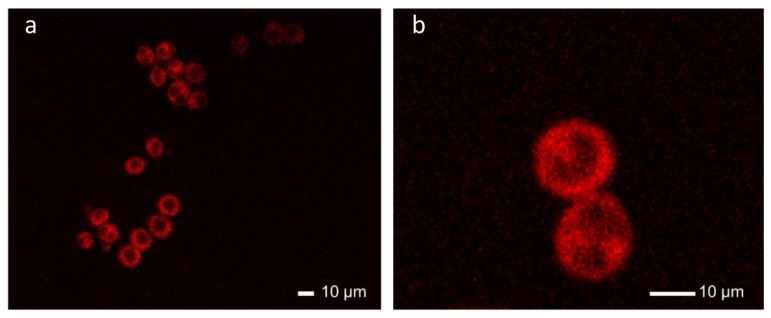
Confocal laser scanning micrographs of live cell macrophages incubated with radical **11**. (**a**) Overview and (**b**) closeup on two macrophages with radical fluorescent dye, indicating that the radical **11** can be utilized for live cell staining.

To test the applicability of our new and water soluble radical fluorescent dye, we incubate macrophages in 500 µM and 50 µM (see Figure 5a,b) solutions of the radical **11** for one hour. The fluorescent cell media is the removed and fresh media is added (see Section 3 for further details). The macrophages are then subjected to confocal laser scanning microscopy, allowing fluorescence imaging of live cells at high resolution. Within the time of incubation, the macrophages have clearly taken up the fluorescent radical dye, which is red fluorescent, when excited with a laser excitation wavelength of *λ* = 405 nm (see Figure 5). The cell nuclei and potential organelles inside of the macrophages appear as dark entities, indicating that our OEG-functionalized radical emitter is too hydrophilic to enter the more hydrophobic compartments of the cell. Over the course of the incubation and imaging, the macrophages appeared healthy, indicating that our radicals are suitable for live cell imaging (see Figure 5). While apoptotic cell rounding is not visible in the total period of 1:15 h of exposure of the cells to the radical dye, its toxicity needs to be further investigated and quantified.

## 3. Materials and Methods

Reagents and solvents are used as commercially provided. Unless otherwise stated, all reactions are performed under a nitrogen atmosphere. Silica gel with a particle size of 40–63 µm is used for column chromatography.

Within the work, 2,2′-((2,6-Dichloro-4-iodophenyl)methylene)bis(1,3,5-trichlorobenzene) (HTTM-I, **3**) and 2,5,8,11-tetraoxatridecan-13-yl methane sulfonate are synthesized, as previously reported [16,25].

**Synthesis of 3-iodo-9-(2,5,8,11-tetraoxatridecan-13-yl]-9*H*-carbazole (1)**

Notably, 2.500 g 3-Iodo-9*H*-carbazole (8.529 mmol, 1 eq.), 0.957 g potassium hydroxide (17.058 mmol, 2 eq.), and 3.688 g sulfonate (12.880 mmol, 1.51 eq.) 2,5,8,11-tetraoxatridecan-13-yl methane are dissolved in 40 mL dimethyl formamide and stirred at room temperature for 24 h. Subsequently, 100 mL water are added, and the mixture is extracted with dichloromethane. The organic layer is washed with water and dried over magnesium sulfate. The solvents are removed under reduced pressure and purification is performed by column chromatography with ethyl acetate. After this, 1.842 g (3.811 mmol, 45%) of **1** are obtained as a light-yellow oil.

**^1^H-NMR (400 MHz, CDCl_3_):** *δ* [ppm] = 8.38 (d, ^4^*J*_H,HH,H_ = 1.8 Hz, 1H), 8.02 (d, ^3^*J*_H,HH,H_ = 7.8 Hz, 1H), 7.70 (dd, ^3^*J*_H,HH,H_ = 8.6, ^4^*J*_H,HH,H_ = 1.8 Hz, 1H), 7.54–7.40 (m, 2H), 7.31–7.26 (m, 1H), 7.25–7.21 (m, 1H), 4.46 (t, ^3^*J*_H,HH,H_ = 5.9 Hz, 2H), 3.85 (t, ^3^*J*_H,HH,H_ = 5.9 Hz, 2H), 3.63–3.54 (m, 2H), 3.59–3.42 (m, 10H), 3.37 (s, 1H).

**^13^C-NMR (100 MHz, CDCl_3_):** *δ* [ppm] = 140.60 (s), 139.94 (s), 133.87 (s), 129.11 (s), 126.44 (s), 125.48 (s), 121.72 (s), 120.50 (s), 119.60 (s), 111.28 (s), 109.15 (s), 81.54 (s), 72.00 (s), 71.09 (s), 70.68 (s), 70.64 (s), 70.56 (s), 69.40 (s), 59.13 (s), 43.40 (s).

**Synthesis of 3-(4,4,5,5-tetramethyl-1,3,2-dioxaborolan-2-yl)-9-(2,5,8,11-tetraoxatridecan-13-yl)-9*H*-carbazole (2)**

Amounts of 700 mg (1.448 mmol, 1 eq.) **1**, 358 mg (1.738 mmol, 1.2 eq.) bis(pinacolato)diboron, 341 mg (3.476 mmol, 2.4 eq.) potassium acetate, and 114 mg (0.145 mmol, 0.1 eq) bis(triphenylphosphine)-palladium(II) dichloride are dissolved in 6 mL dimethyl formamide The reaction solution is stirred at 80 °C for 18 h. After cooling to room temperature, the mixture is extracted with ethyl acetate. The organic layer is washed with brine and dried over magnesium sulfate. The solvents are removed under reduced pressure. The crude product is purified by silica column chromatography using an ethyl acetate/petroleum ether 2:1 mixture as the eluent. Additionally, 0.212 g (0.438 mmol, 30%) **2** are obtained as a light-yellow oil.

**^1^H NMR (600 MHz, CDCl_3_):** *δ* [ppm] = 8.57 (s, 1H), 8.09 (d, ^3^*J*_H,HH,H_ = 7.8 Hz, 1H), 7.89 (d, ^3^*J*_H,HH,H_ = 8.3 Hz, 1H), 7.45–7.35 (m, 3H), 7.26–7.15 (m, 1H), 4.43 (t, ^3^*J*_H,HH,H_ = 5.9 Hz, 2H), 3.80 (t, ^3^*J*_H,HH,H_ = 6.0 Hz, 2H), 3.52 (t, ^3^*J*_H,HH,H_ = 4.4 Hz, 2H), 3.45 (d, ^3^*J*_H,HH,H_ = 4.4 Hz, 11H), 3.31 (s, 3H), 1.37 (s, 12H).

**Synthesis of 3-(4-(bis(2,4,6-trichlorophenyl)methyl)-3,5-dichlorophenyl)-9-(2,5,8,11-tetraoxatri- decan-13-yl)-9*H*-carbazole (4)**

Amounts of 60 mg (0.093 mmol, 1 eq.) HTTM-I (**3**), 67 mg (0.139 mmol, 1.5 eq.) **2**, and 257 mg (1.209 mmol, 13 eq.) potassium phosphate are dissolved in a mixture of 2 mL toluene, 1 mL water, and 0.5 mL ethanol, as well as 16 mg (0.014 mmol, 0.15 eq.) Tetrakis(triphenylphosphine)palladium(0) are added and the mixture is stirred for 48 h at 90 °C. After cooling to room temperature, the reaction mixture is extracted with dichloromethane. The organic phase is dried over magnesium sulfate and the solvents are removed under reduced pressure. The crude product is purified by silica column chromatography using a petroleum ether/ethyl acetate 1:1 mixture as the eluent to give 65 mg (0.074 mmol, 80%) **4** as a colorless solid.

**HRMS (MALDI):** *m*/*z* = 874.9855 [M]^+^; calc. *m*/*z* = 874.9859.

**^1^H NMR (600 MHz, CDCl_3_):** *δ* [ppm] = 8.28 (d, ^4^*J*_H,HH,H_ = 2.0 Hz, 1H), 8.12 (d, ^3^*J*_H,HH,H_ = 7.9 Hz, 1H), 7.70 (d, *J* = 2.1 Hz, 1H), 7.67 (dd, ^3^*J*_H,HH,H_ = 8.5, ^4^*J*_H,HH,H_ = 1.9 Hz, 1H), 7.57 (d, ^4^*J*_H,HH,H_ = 2.1 Hz, 1H), 7.53 (d, ^3^*J*_H,HH,H_ = 8.5 Hz, 1H), 7.50–7.47 (m, 2H), 7.41–7.35 (m, 2H), 7.31–7.26 (m, 3H), 6.80 (s, 1H), 4.53 (t, ^3^*J*_H,HH,H_ = 6.0 Hz, 2H), 3.90 (t, ^3^*J*_H,HH,H_ = 5.9 Hz, 2H), 3.64–3.53 (m, 2H), 3.55–3.46 (m, 10H), 3.34 (s, 3H).

**^13^C-NMR (151 MHz, CDCl_3_):** *δ* [ppm] = 143.10 (s), 141.26 (s), 140.96 (s), 138.39 (s), 138.13 (s), 137.72 (s), 137.44 (s), 137.31 (s), 137.09 (s), 134.69 (s), 134.65 (s), 133.69 (s), 133.67 (s), 132.50 (s), 130.18 (s), 130.09 (s), 128.89 (s), 128.57 (s), 128.54 (s), 128.50 (s), 126.88 (s), 126.33 (s), 124.79 (s), 123.69 (s), 123.03 (s), 120.58 (s), 119.66 (s), 118.84 (s), 109.69 (s), 109.35 (s), 72.06 (s), 71.17 (s), 70.77 (s), 70.74 (s), 70.71 (s), 70.62 (s), 69.52 (s), 59.17 (s), 50.20 (s), 43.57 (s).

**Synthesis of radical (9)**

An amount of 43 mg (0.049 mmol, 1 eq.) **4** is dissolved in 2 mL THF, and 0.25 mL (0.245 mmol, 5 eq.) of a 1M solution of tetrabutylammonium hydroxide in methanol are added. The mixture is stirred overnight at room temperature and is then treated with 36 mg (0.147 mmol, 3 eq.) of *p*-Chloranil. The reaction is continued for 2 h in the dark. After removal of the solvent, the crude product is purified via column chromatography using a petroleum ether/ethyl acetate 1:1 mixture to give 34 mg (0.039 mmol, 79%) **9** as a green solid.

**HRMS (MALDI):** *m*/*z* = 874.9868 [M-H]^+^; calc. *m*/*z* = 874.9859.

**EPR (toluene):** *g* = 2.0036.

**Synthesis of ((4-(bis(2,4,6-trichlorophenyl)methyl)-3,5-dichlorophenyl)ethynyl)trimethylsilane (5)**

Amounts of 500 mg (0.774 mmol, 1 eq.) HTTM-I (**3**), 15 mg (0.077 mmol, 0.1 eq.) copper(I) iodide, and 89 mg (0.077 mmol, 0.1 eq.) tetrakis(triphenylphosphine)palladium(0) are dissolved in 10 mL of a 2:1 tetrahydrofuran/triethylamine mixture. After 15 min., 0.17 mL (1.161 mmol, 1.5 eq.) trimethylsilyl acetylene are added, and the reaction is continued at 60 °C for 16 h. After cooling to room temperature, the reaction mixture is filtrated, and the volatiles are removed under reduced pressure. After column chromatography, using petroleum ether as an eluent, 415 mg (0.674 mmol, 87%) **5** are obtained as a colorless solid.

**^1^H NMR (600 MHz, CDCl_3_):** *δ* [ppm] = 7.43 (d, ^4^*J*_H,HH,H_ = 1.7 Hz, 1H), 7.35 (m, ^4^*J*_H,HH,H_ = 2.1 Hz, 2H), 7.30 (d, ^4^*J*_H,HH,H_ = 1.8 Hz, 1H), 7.24–7.21 (m, 2H), 6.69 (s, 1H), 0.24 (s, 9H).

**^13^C-NMR (151 MHz, CDCl_3_):** *δ* [ppm] = 138.15 (s), 138.06 (s), 137.33 (s), 137.28 (s), 137.25 (s), 136.59 (s), 135.71 (s), 134.17 (s), 133.87 (s), 133.85 (s), 133.30 (s), 131.59 (s), 130.12 (s), 128.56 (s), 128.54 (s), 124.36 (s), 101.74 (s), 98.10 (s), 50.30 (s), −0.13 (s).

**Synthesis of 2,2′-((2,6-dichloro-4-ethynylphenyl)methylene)bis(1,3,5-trichlorobenzene) (6)**

The cleavage of the TMS-group is performed under ambient conditions without a nitrogen atmosphere. Additionally, 400 mg (0.649 mmol, 1 eq.) **5** are dissolved in a 3:1 dichloromethane/methanol mixture and 900 mg (6.512 mmol, 10 eq.) potassium carbonate are added. The mixture is stirred for 3 h at room temperature and is then filtrated. The filtrate is washed with water and dried over magnesium sulfate. The solvent is removed to give 350 mg (0.644 mmol, 99%) **6** as a pale beige solid. at

**^1^H NMR (400 MHz, CDCl_3_):** *δ* [ppm] = 7.45 (d, ^4^*J*_H,HH,H_ = 1.8 Hz, 1H), 7.38–7.34 (m, 2H), 7.32 (d, ^4^*J*_H,HH,H_ = 1.8 Hz, 1H), 7.24–7.21 (m, 2H), 6.71 (s, 1H), 3.17 (s, 1H).

**^13^C-NMR (151 MHz, CDCl_3_):** *δ* [ppm] = 138.12 (s), 138.08 (s), 137.46 (s), 137.28 (s), 137.26 (s), 136.69 (s), 136.22 (s), 134.09 (s), 133.93 (s), 133.91 (s), 133.52 (s), 131.79 (s), 130.15 (s), 130.13 (s), 128.57 (s), 123.36 (s), 80.68 (s), 80.28 (s), 50.31 (s).

**Synthesis of 2-(2-(2-(2-(4-(4-(bis(2,4,6-trichlorophenyl)methyl)-3,5-dichlorophenyl)-1H-1,2,3-triazol-1-yl)ethoxy)ethoxy)ethoxy)ethan-1-ol (7)**

Amounts of 120 mg (0.221 mmol, 1.0 eq.) **6** and 48 mg (0.219 mmol, 1 eq.) azido-PEG4-alcohol are dissolved in 2 mL DMF and 0.03 mL (0.22 mmol, 1 eq.) triethylamine. Additionally, 13 mg (0.066 mmol, 0.3 eq.) Copper(I) iodide are added, and the mixture is stirred for 24 h at room temperature. After removal of the solvents purification via column chromatography is carried out with an ethyl acetate/methanol 10:1 mixture to give 152 mg (0.199 mmol, 90%) **7** as a colorless solid.

**HRMS (MALDI):** *m*/*z* = 785.9179 [M + Na]^+^; calc. *m*/*z* = 785.9192.

**^1^H NMR (600 MHz, CDCl_3_):** *δ* [ppm] = 8.13 (s, 1H), 7.87 (d, ^4^*J*_H,HH,H_ = 1.9 Hz, 1H), 7.72 (d, ^4^*J*_H,H_ = 1.8 Hz, 1H), 7.38–7.33 (m, 2H), 7.24 (d, ^4^*J*_H,H_ = 2.3 Hz, 1H), 7.23 (d, ^4^*J*_H,H_ = 2.3 Hz, 1H), 6.75 (s, 1H), 4.68–4.51 (m, 2H), 3.94–3.80 (m, 2H), 3.73–3.56 (m, 13H).

**^13^C-NMR (151 MHz, CDCl_3_):** *δ* [ppm] = 145.18 (s), 138.45 (s), 138.31 (s), 138.14 (s), 137.55 (s), 137.49 (s), 134.65 (s), 134.61 (s), 134.47 (s), 133.98 (s), 133.96 (s), 132.34 (s), 130.35 (s), 130.31 (s), 128.77 (s), 128.73 (s), 127.36 (s), 125.75 (s), 122.44 (s), 72.76 (s), 70.89 (s), 70.87 (s), 70.72 (s), 70.55 (s), 69.82 (s), 62.04 (s), 50.86 (s), 50.46 (s).

**Synthesis of radical (10)**

Amounts of 52 g (0.068 mmol, 1 eq.) **7** are dissolved in 2 mL anhydrous THF and 0.34 mL (0.340 mmol, 5 eq.) of a 1M solution of tetrabutylammonium hydroxide in methanol are added. The solution is stirred overnight at room temperature before adding 50 g *p*-chloranil (0.204 mmol, 3 eq.). The reaction is continued for further 2 h under light exclusion. The solvents are removed under reduced pressure, and purification via column chromatography is carried out with an ethyl acetate/methanol 10:1 mixture to give 31 mg (0.049 mmol, 72%) **10** as a red solid.

**HRMS (MALDI):** *m*/*z* = 785.9184 [M-H + Na]^+^; calc. *m*/*z* = 785.9192.

**EPR (toluene):** *g* = 2.0036.

**Synthesis of 59-(4-(4-(bis(2,4,6-trichlorophenyl)methyl)-3,5-dichlorophenyl)-1H-1,2,3-triazol-1-yl)-3,6,9,12,15,18,21,24,27,30,33,36,39,42,45,48,51,54,57-nonadecaoxanonapentacontan-1-ol (8)**

Amounts **of** 100 mg **6** (0.184 mmol, 1.0 eq.) and 170 mg (0.184 mmol, 1 eq.) azido-PEG20-alcohol (95% oligomer purity) are dissolved in 2 mL DMF with 0.03 mL (0.18 mmol, 1 eq.) triethylamine. After the addition of 11 mg (0.055 mmol, 0.3 eq.) copper(I) iodide, the reaction is stirred at room temperature for 20 h. The solvents are removed under reduced pressure, and purification is performed via column chromatography using an ethyl acetate/methanol 5:1 mixture to give 243 mg (0.166 mmol, 90%) **8** as a colorless oil.

**HRMS (MALDI):** *m*/*z* = 1490.3397 [M + Na]^+^; calc. *m*/*z* = 1490.3387.

**^1^H NMR (600 MHz, CDCl_3_):** *δ* [ppm] = 8.10 (s, 1H), 7.85 (d, ^4^*J*_H,H_ = 1.9 Hz, 1H), 7.71 (d, ^4^*J*_H,H_ = 1.8 Hz, 1H), 7.37–7.34 (m, 2H), 7.24 (d, ^4^*J*_H,H_ = 2.3 Hz, 1H), 7.23 (d, ^4^*J*_H,H_ = 2.3 Hz, 1H), 6.74 (s, 1H), 4.61–4.57 (m, 2H), 3.92–3.87 (m, 2H), 3.74–3.69 (m, 2H), 3.68–3.48 (m, 75H).

**^13^C-NMR (151 MHz, CDCl_3_):** *δ* [ppm] = 144.89 (s), 138.22 (s), 138.08 (s), 137.90 (s), 137.32 (s), 137.26 (s), 137.25 (s), 134.43 (s), 134.39 (s), 134.21 (s), 133.74 (s), 133.72 (s), 132.14 (s), 130.12 (s), 130.07 (s), 128.54 (s), 128.50 (s), 127.12 (s), 125.51 (s), 122.16 (s), 72.64 (s), 70.82–70.49 (m), 70.40 (s), 69.49 (s), 61.79 (s), 50.65 (s), 50.23 (s).

**Synthesis of radical (11)**

Amounts of 70 mg (0.048 mmol, 1 eq.) **8** are dissolved in 1 mL anhydrous THF and 0.24 mL (0.238 mmol, 5 eq.) of a 1M solution of tetrabutylammonium hydroxide in methanol are added. The solution is stirred overnight at room temperature and 24 mg (0.143 mmol, 3 eq.) silver nitrate are added. After stirring for 2 h in the dark, the reaction mixture is extracted with dichloromethane and washed with water. Purification is performed via reversed phase HPLC (acetonitrile/water = 70:30–95:5) to give **11** as a red oil. Since the purification is performed in portions of 1 mg, the yield is not determined because of substantial losses during the chromatography procedure.

**HRMS (APCI):** *m*/*z* = 1468.3449 [M-H + H]^+^ calc. *m*/*z* = 1468.3523.

**EPR (toluene):** *g* = 2.0036.

**Cell culture:** The J774A.1 mouse macrophage cell line was cultivated in DMEM medium (Sigma-Aldrich, Schnelldorf, Germany) with 1mM sodium pyruvate (Sigma-Aldrich), 4 mM l-glutamine (Sigma-Aldrich), 100 U/mL penicillin (Sigma-Aldrich), 0.1 mg/mL streptomycin (Sigma-Aldrich), 10% FCS (Gibco, Carlsbad, CA, USA) at 37 °C and 5% CO_2_. One day prior the experiment, 10,000 cells were seeded into a 8 well chamber slide. The cells were washed with phenol red-free DMEM (with 1 mM sodium pyruvate, 4 mM l-glutamine, 100 U/mL penicillin, 0.1 mg/mL streptomycin, 10% FCS) and 200 µL of a solution of **9** (50 µM and 500 µM, respectively, dissolved in the phenol red-free DMEM) was added. After one hour in the cell culture incubator, the cells were washed with phenol red-free DMEM and the uptake was recorded by using a Leica TCS SP 8 confocal microscope (Leica Microsystems, Wetzlar, Germany).

**Quantum chemical calculations:** DFT and TD-DFT calculations were performed with Gaussian g16.C.01.2 [26]. The radicals were optimized in their ground state geometry employing uPBE0-GD3(BJ)/6-311++G(d,p) with the SCRF-SMD method to mimic a water environment [27,28,29]. The vibrational frequencies were calculated to verify the structures as minima. Vertical excitations were obtained from TD-DFT single-point calculations on the above-described level of theory for the molecules in their ground-state geometry. Natural transition orbitals were visualized with Gaussview 6.1.5 [30].

**HPLC:** Reversed phase high-performance liquid chromatography was performed on a Thermo Scientific (Waltham, MA, USA) UltiMate 3000 using an Agilent (Santa Clara, CA, USA) Eclipse Plus C18 column with a particle size of 3.5 µm.

**NMR:** ^1^H and ^13^C nuclear magnetic resonance spectra were recorded with a Bruker (Billerica, MA, USA) AVANCE NEO 400 or AVANCE NEO 600 from solutions in deuterated chloroform.

**EPR:** X-Band electron paramagnetic resonance spectra were measured for toluene solutions of the radicals with a Bruker e-scan EPR spectrometer at ambient temperature. Landé g-factors were determined using 2,2-diphenyl-1-picrylhydrazyl (DPPH) as a reference.

**Mass spectrometry:** Atmospheric pressure chemical ionization (APCI) and matrix-assisted laser desorption/ionization (MALDI) high-resolution mass spectrometry (HRMS) were performed on a Bruker solariX Fourier transform ion cyclotron resonance (FTICR) mass spectrometer using trans-2-[3-(4-tert-Butyl-phenyl)-2-methyl-2-propenylidene] malononitrile (DCTB) as a matrix for MALDI.

**UV/vis and photoluminescence spectroscopy:** Ultraviolet/visible spectra were recorded using a Perkin Elmer (Waltham, MA, USA) Lambda 365 spectrophotometer and photoluminescence spectra with a PerkinElmer FL6500. Fluorescence quantum yields *ϕ* were determined using a Hamamatsu Quantaurus-QY (C11347) (Hamamatsu Photonics, Herrsching am Ammersee, Germany) for toluene and water solutions.

**DLS:** Dynamic light scattering experiments were performed on a Nano-Zetasizer (Malvern Instruments, Westborough, MA, USA) at room temperature under a scattering angle of 173° at *λ* = 632.8 nm.

## 4. Conclusions

Our study of OEG functionalized TTM radicals shows that LE states are favourable for light emission in aqueous media. OEGylation appears as a suitable strategy to induce water solubility without affecting the optical or electronic properties of the light emitting radicals. The OEG-functionalized fluorescent radical dye construct is suitable as a water soluble marker for live cell imaging in cell culture. In the future, LE radical emitters with greater quantum yields might further improve the cell imaging capability of this new class of cellular imaging dyes. Moreover, the impact on the cells and the stability of the radical and fluorescence performance needs to be elucidated. In principle, these new water soluble radical dyes would enable dual mode imaging, where the free electron could be employed as a spin lable, especially when considering using such water soluble radical dyes for in vivo experiments.

## Data Availability

Data are contained within the article and Supplementary Materials.

## Supplementary Materials

The following supporting information can be downloaded at: https://www.mdpi.com/article/10.3390/molecules29050995/s1, NMR spectra of new closed-shell compounds, X-band EPR spectra of new radicals, geometries obtained by DFT calculations, Lippert–Mataga plots, DLS measurements.
