# Supplementary material for: Water-Soluble Trityl Radicals for Fluorescence Imaging
Source: Molecules. 2024 Feb 25;29(5):995. doi: 10.3390/molecules29050995 (PMC10934868; doi:10.3390/molecules29050995)
Supplement: Supplementary file 1 [file molecules-29-00995-s001.zip › molecules-2881493-supplementary.pdf]

Supporting Information

Water-Soluble Trityl Radicals for Fluorescence Imaging

Mona E. Arnold,[1] Larissa Schoeneburg,[1] Markus Lamla,[1] and Alexander J.C. Kuehne[1]*

[1]Institute of Organic and Macromolecular Chemistry, Ulm University, Albert-Einstein-Allee 11, 89081 Ulm, Germany.

Contents

1. NMR Spectra of New Closed-Shell Compounds

2. X-Band EPR Spectra of New Radicals

3. Geometries obtained by DFT Calculations

4. Lippert-Mataga Plots

5. DLS Measurements

1. NMR Spectra of New Closed-Shell Compounds

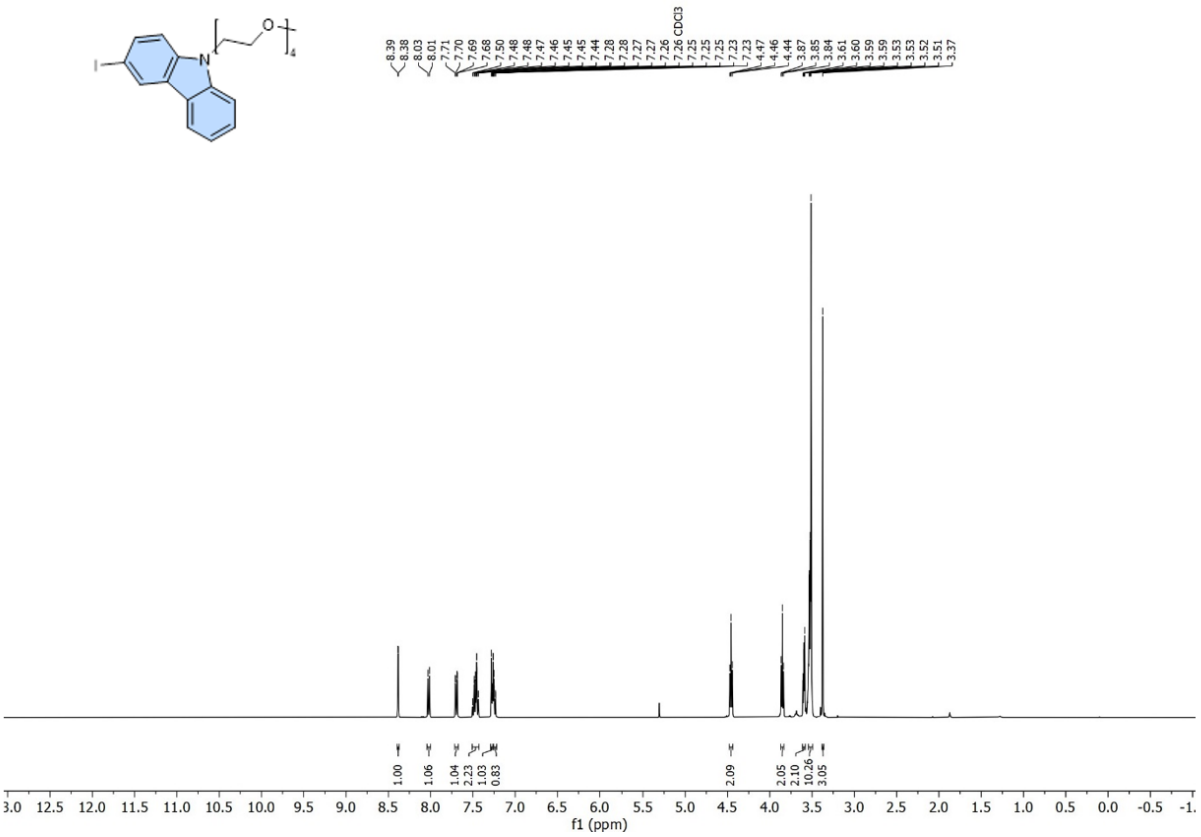

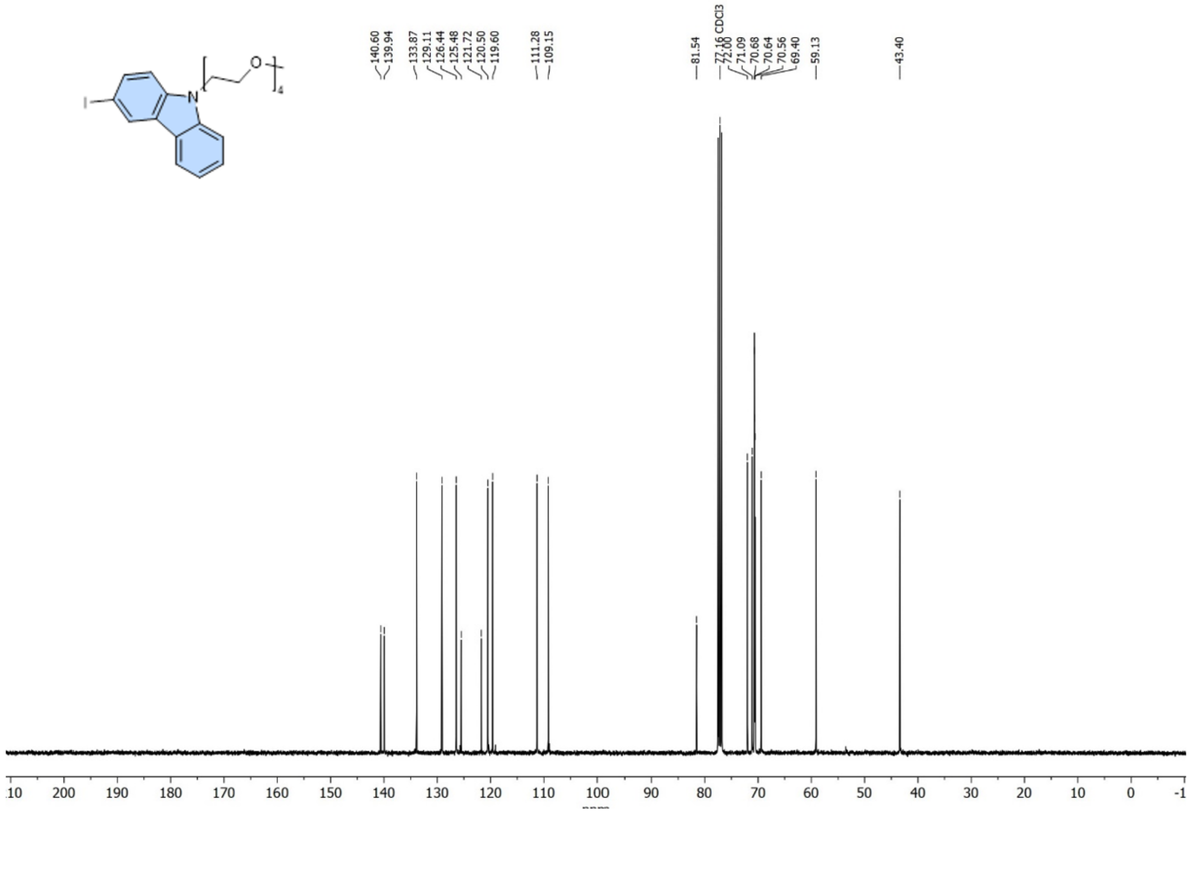

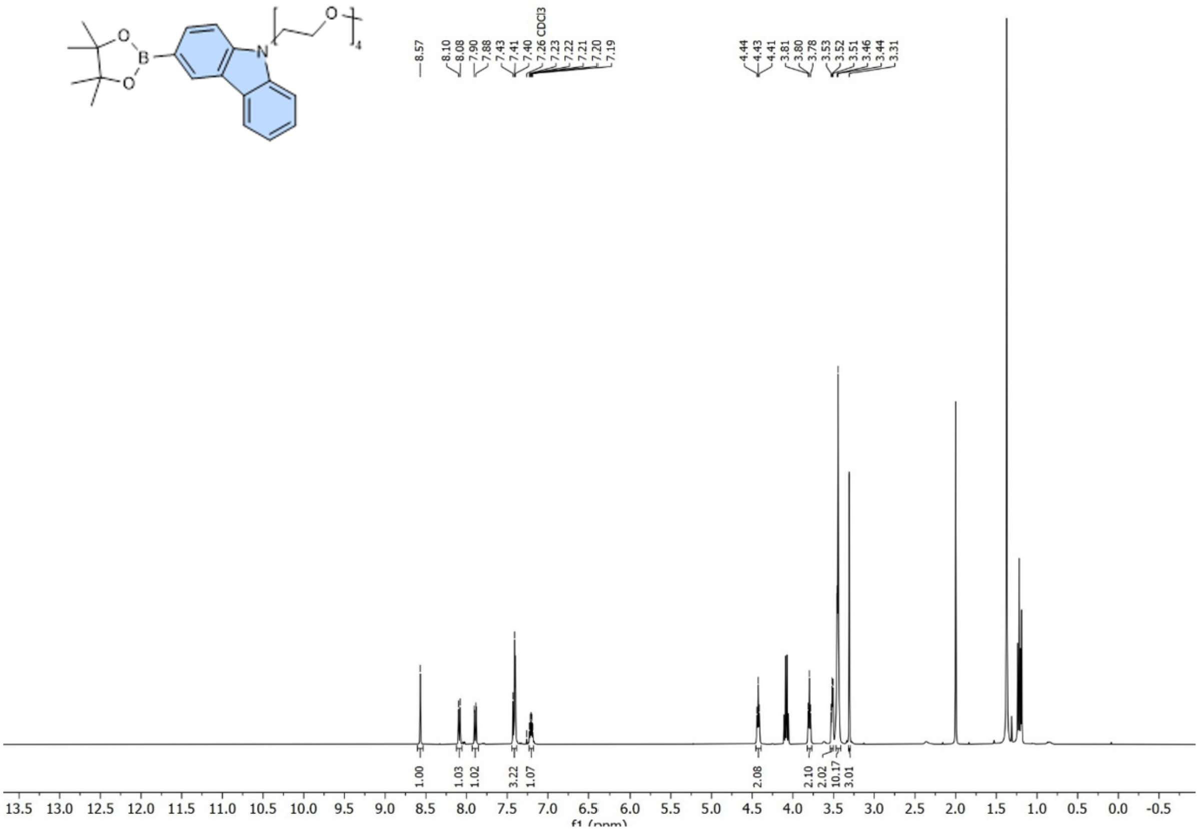
O
B
N
4
8.57
8.10
8.08
7.90
7.88
7.43
7.41
7.40
7.26 CDCl3
7.23
7.22
7.21
7.20
7.19
4.44
4.43
4.41
3.81
3.80
3.78
3.53
3.52
3.51
3.46
3.44
3.31
1.00
1.03
1.02
3.22
1.07
2.08
2.10
2.02
10.17
3.01
f1 (ppm)

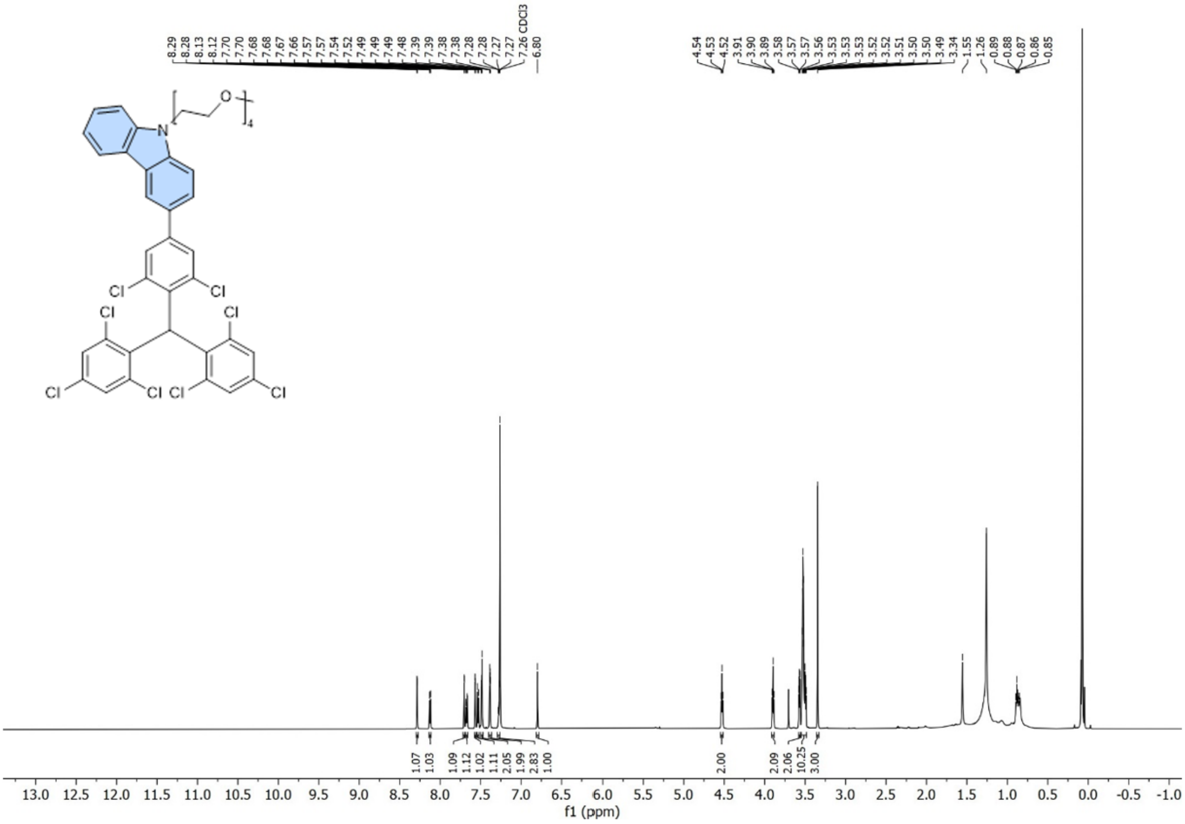
8.29
8.28
8.13
8.12
7.70
7.70
7.68
7.68
7.67
7.66
7.57
7.57
7.54
7.52
7.49
7.49
7.49
7.48
7.39
7.39
7.38
7.38
7.28
7.28
7.27
7.26 CDCl3
6.80
4.54
4.53
4.52
3.91
3.90
3.89
3.58
3.57
3.56
3.53
3.53
3.53
3.52
3.52
3.51
3.50
3.50
3.49
3.34
1.55
1.26
0.89
0.88
0.87
0.86
0.85
N
O
4
Cl
1.07
1.03
1.09
1.12
1.02
1.11
2.05
1.99
2.83
1.00
2.00
2.09
2.06
10.25
3.00
f1 (ppm)

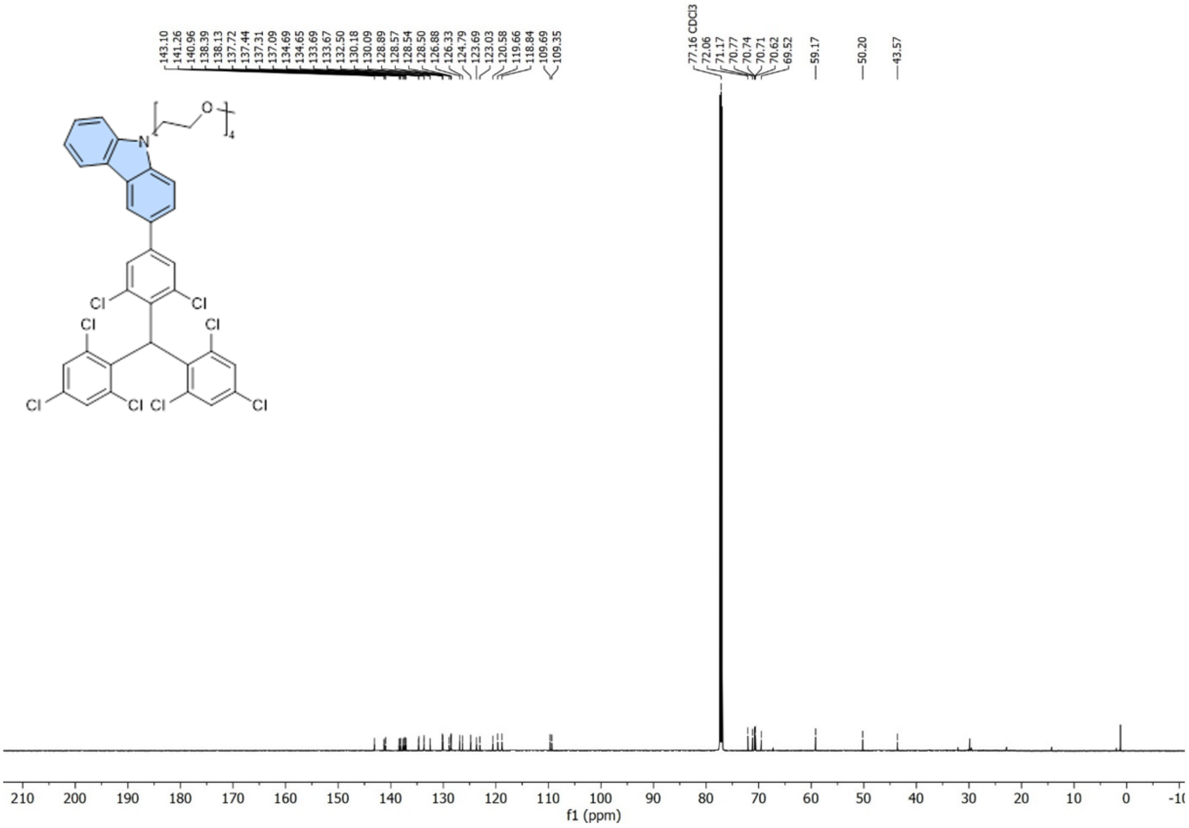
143.10
141.26
140.96
138.39
138.13
137.72
137.44
137.31
137.09
134.69
134.65
133.69
133.67
132.50
130.18
130.09
128.89
128.57
128.54
128.50
126.88
126.33
124.79
123.69
123.03
120.58
119.66
118.84
109.69
109.35
77.16 CDCl3
72.06
71.17
70.77
70.74
70.71
70.62
69.52
59.17
50.20
43.57
N
O
4
Cl
f1 (ppm)

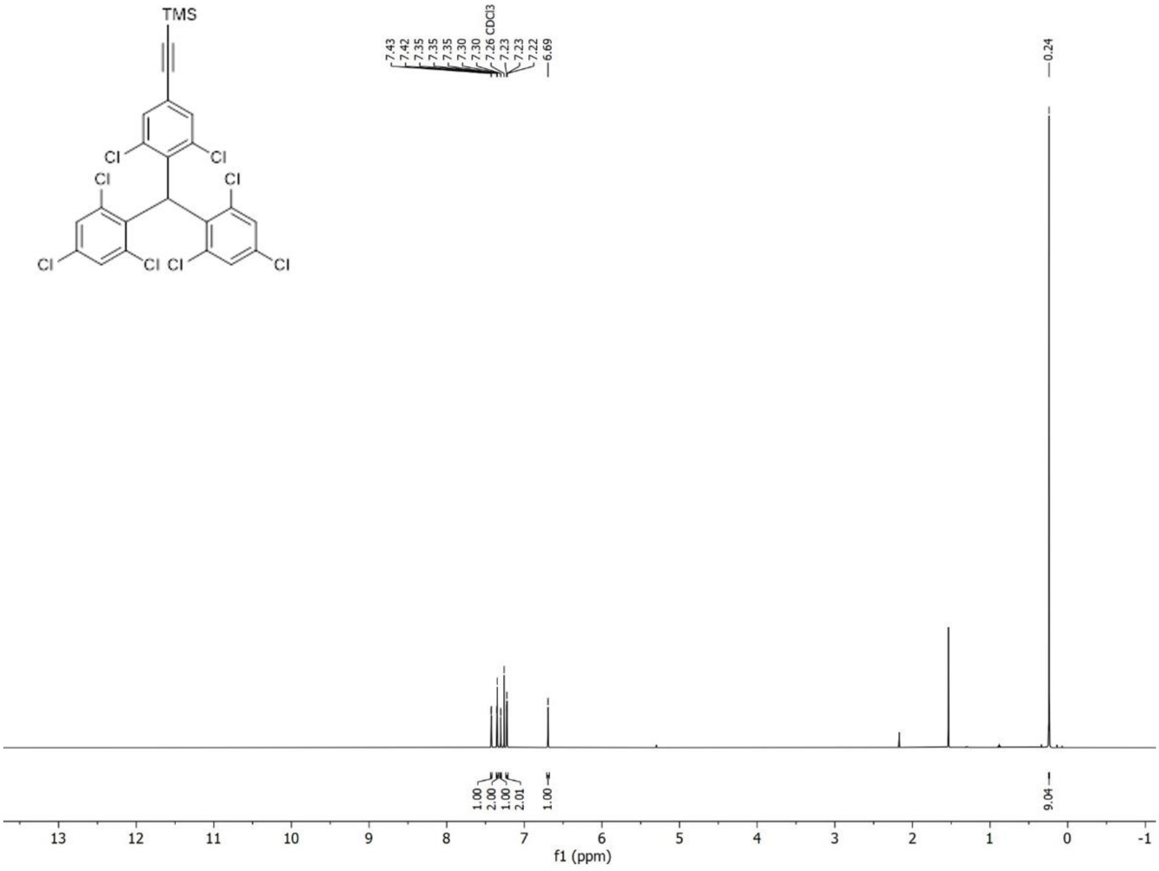
TMS
Cl
7.43
7.42
7.35
7.35
7.35
7.30
7.30
7.26 CDCl3
7.23
7.23
7.22
6.69
0.24
1.00
2.00
1.00
2.01
1.00
9.04
f1 (ppm)

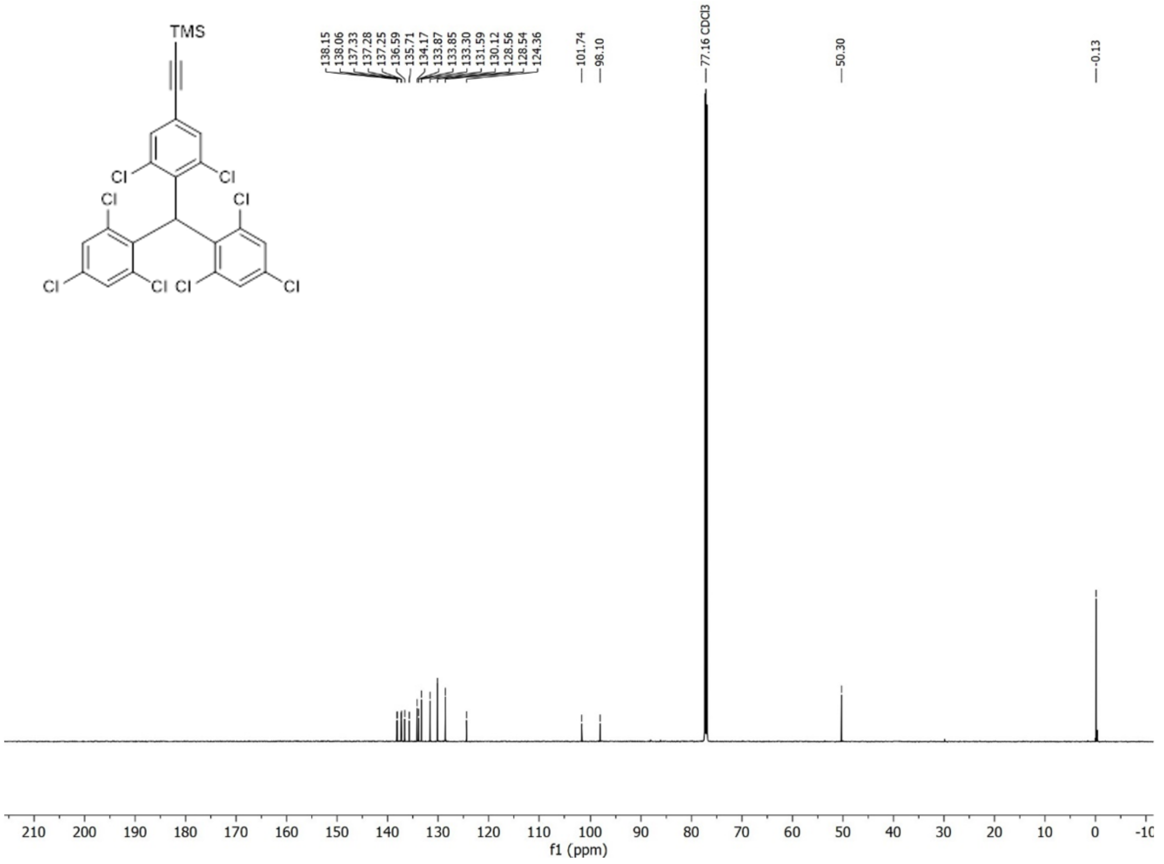
TMS
Cl
138.15
138.06
137.33
137.28
137.25
136.59
135.71
134.17
133.87
133.85
133.30
131.59
130.12
128.56
128.54
124.36
101.74
98.10
77.16 CDCl3
50.30
-0.13
f1 (ppm)

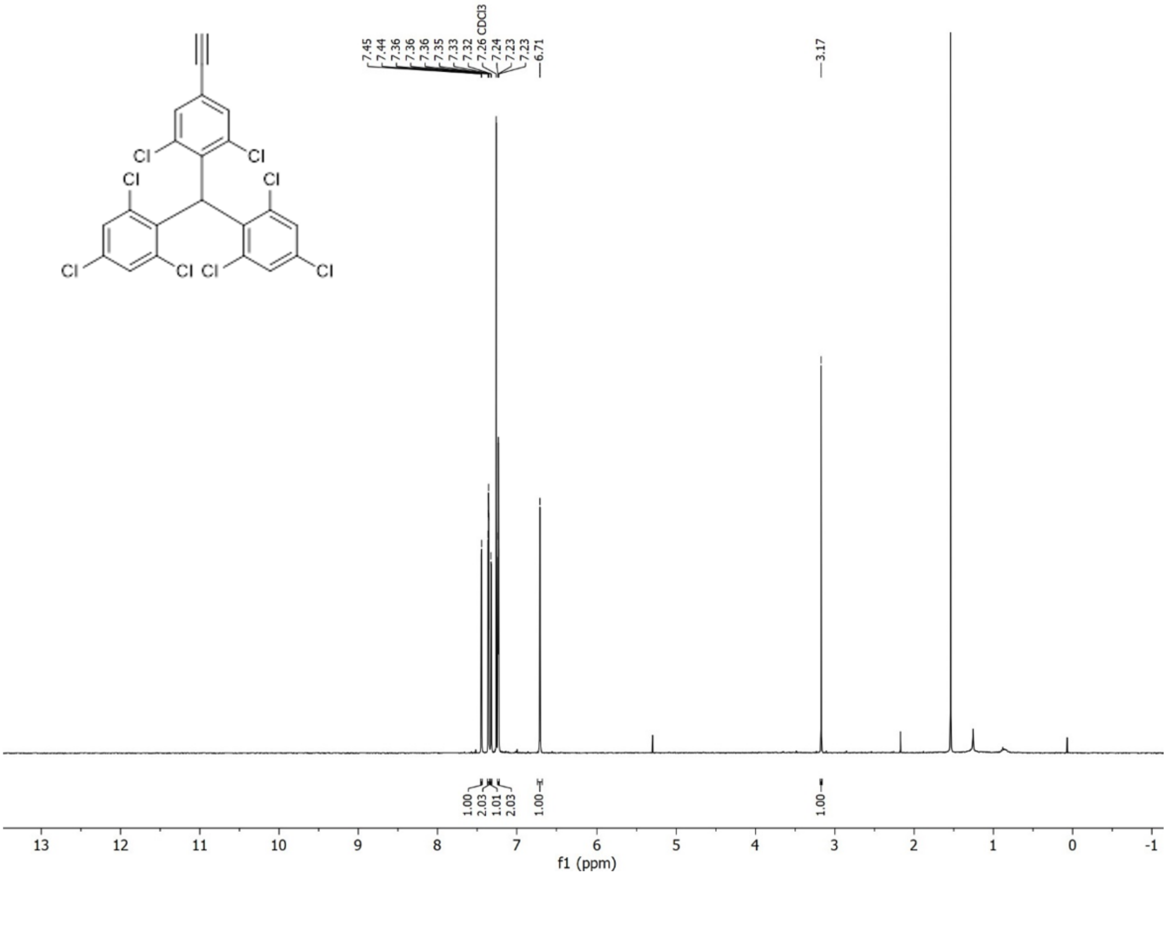
Cl
7.45
7.44
7.36
7.36
7.36
7.35
7.33
7.32
7.26 CDCl3
7.24
7.23
7.23
6.71
3.17
1.00
2.03
1.01
2.03
1.00
1.00
f1 (ppm)

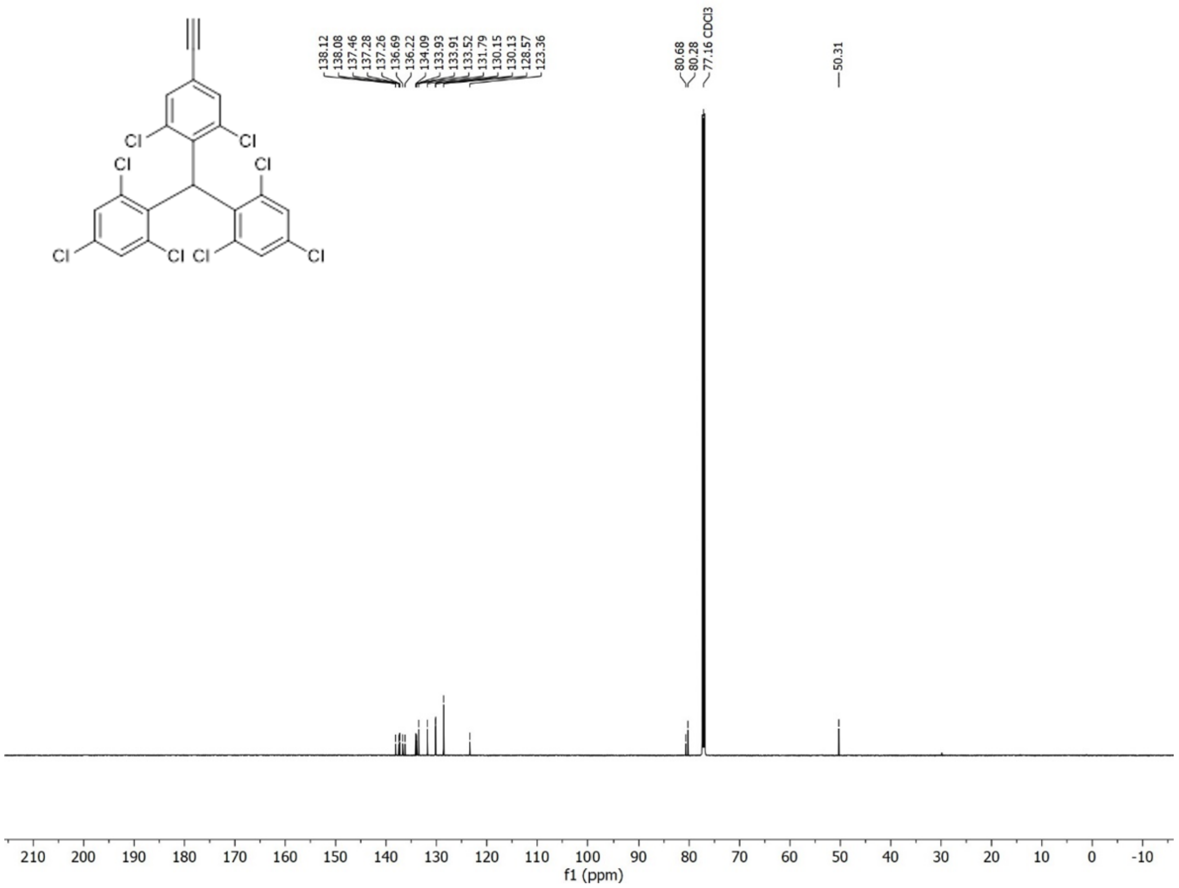

138.12
138.08
137.46
137.28
137.26
136.69
136.22
134.09
133.93
133.91
133.52
131.79
130.15
130.13
128.57
123.36
80.68
80.28
77.16 CDCl3
50.31
Cl
f1 (ppm)

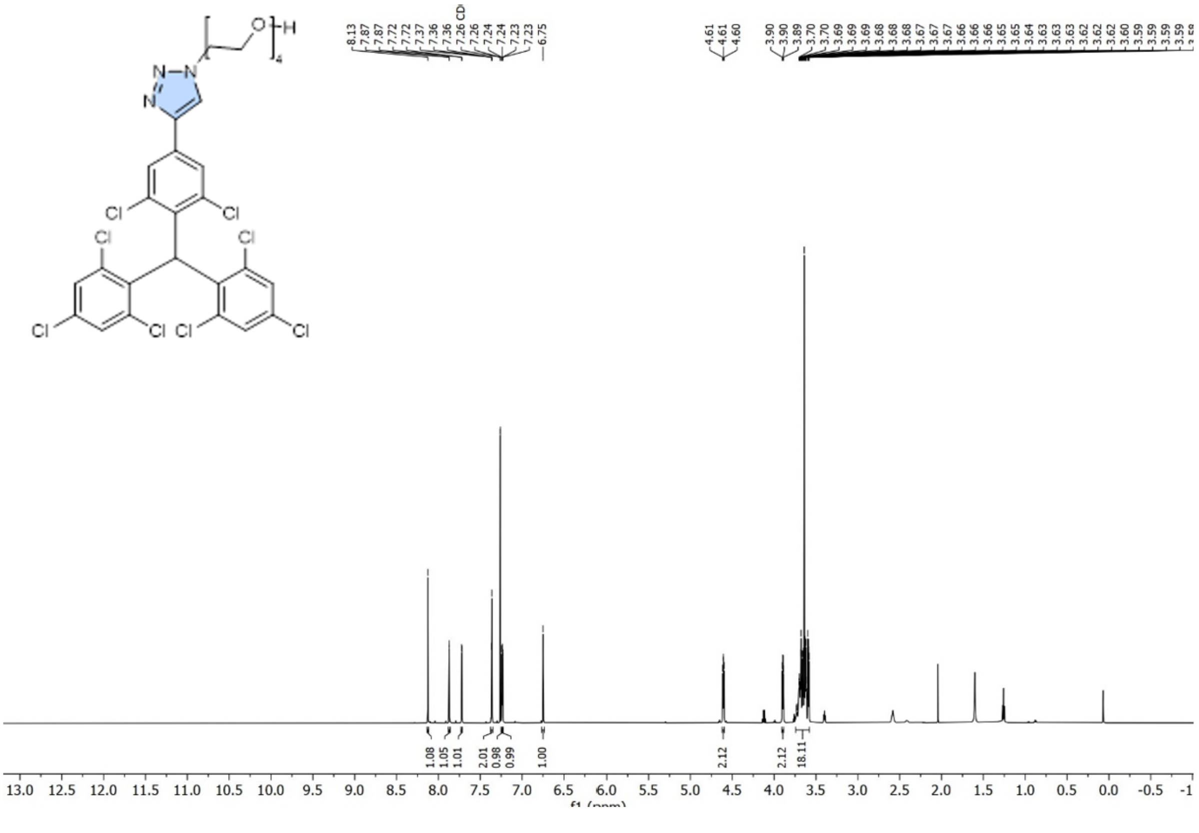

8.13
7.87
7.72
7.72
7.37
7.36
7.36
7.26 CD
7.26
7.24
7.24
7.23
7.23
6.75
4.61
4.61
4.60
3.90
3.90
3.89
3.70
3.69
3.69
3.69
3.68
3.68
3.68
3.67
3.67
3.66
3.66
3.65
3.65
3.64
3.63
3.63
3.63
3.62
3.62
3.60
3.59
3.59
3.59
3.59
1.08
1.05
1.01
2.01
0.98
0.99
1.00
2.12
2.12
18.11
Cl
N
H
4

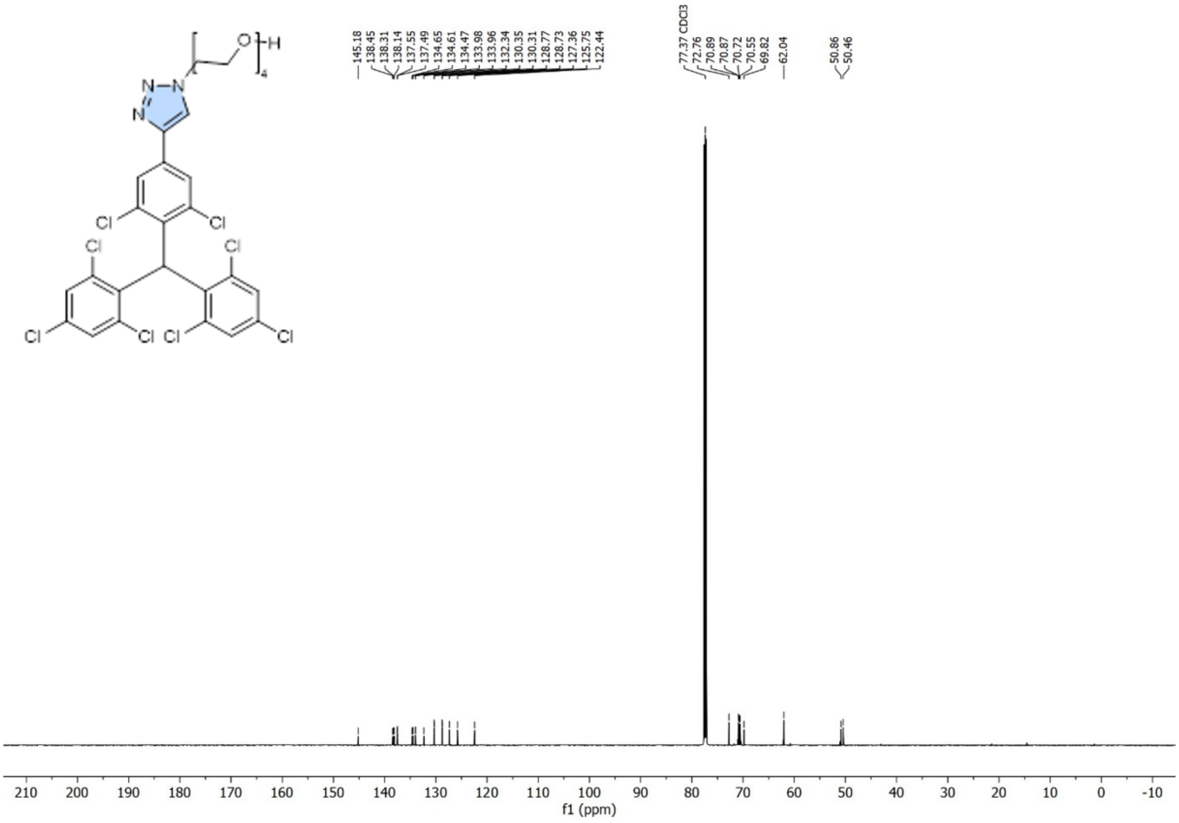
N
N
N
O
H
4
Cl
Cl
Cl
Cl
Cl
Cl
Cl
Cl
145.18
138.45
138.31
138.14
137.55
137.49
134.65
134.61
134.47
133.98
133.96
132.34
130.35
130.31
128.77
128.73
127.36
125.75
122.44
77.37 CDCl3
72.76
70.89
70.87
70.72
70.55
69.82
62.04
50.86
50.46
210
200
190
180
170
160
150
140
130
120
110
100
90
80
70
60
50
40
30
20
10
0
-10
f1 (ppm)

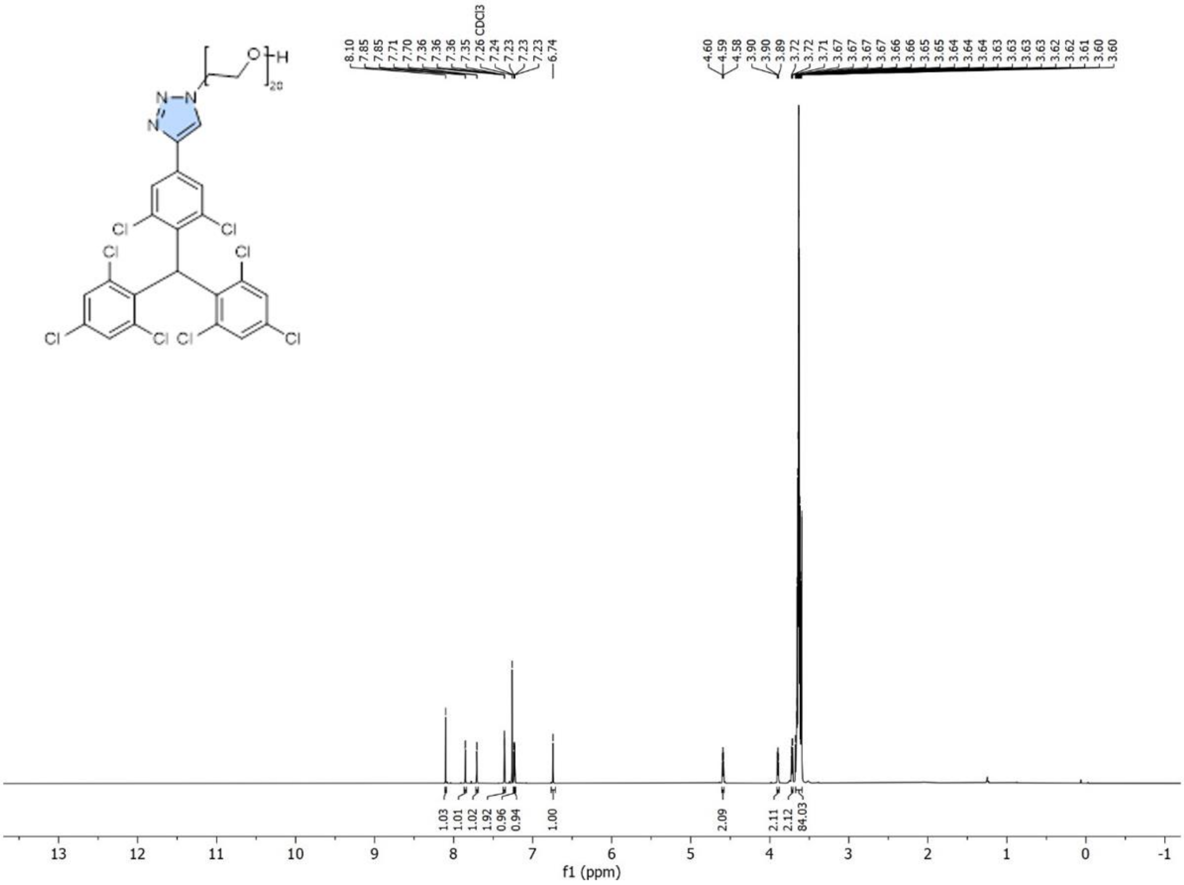
N
N
N
O
H
20
Cl
Cl
Cl
Cl
Cl
Cl
Cl
Cl
8.10
7.85
7.85
7.71
7.70
7.36
7.36
7.36
7.35
7.26 CDCl3
7.24
7.23
7.23
7.23
6.74
4.60
4.59
4.58
3.90
3.90
3.89
3.72
3.72
3.71
3.67
3.67
3.67
3.67
3.66
3.66
3.65
3.65
3.64
3.64
3.64
3.63
3.63
3.63
3.63
3.62
3.62
3.61
3.60
3.60
1.03
1.01
1.02
1.92
0.96
0.94
1.00
2.09
2.11
2.12
84.03
13
12
11
10
9
8
7
6
5
4
3
2
1
0
-1
f1 (ppm)

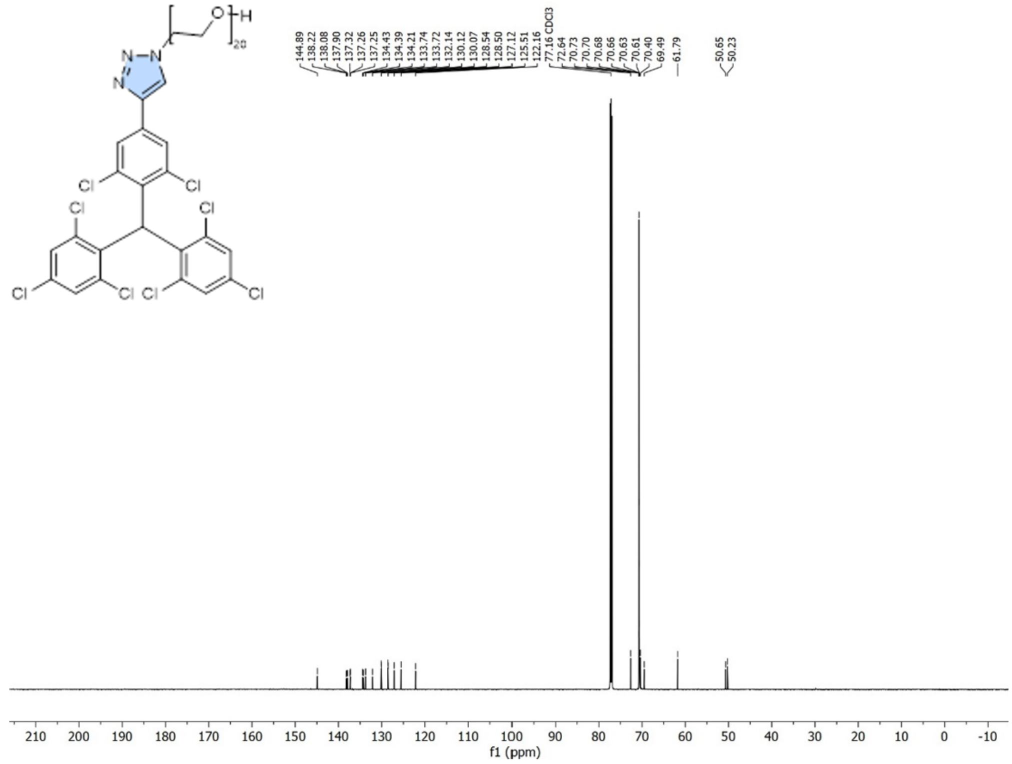

Figure S1: ^{1}H- and ^{13}C-Spectra of new closed-shell compounds.

2. X-Band EPR Spectra of New Radicals

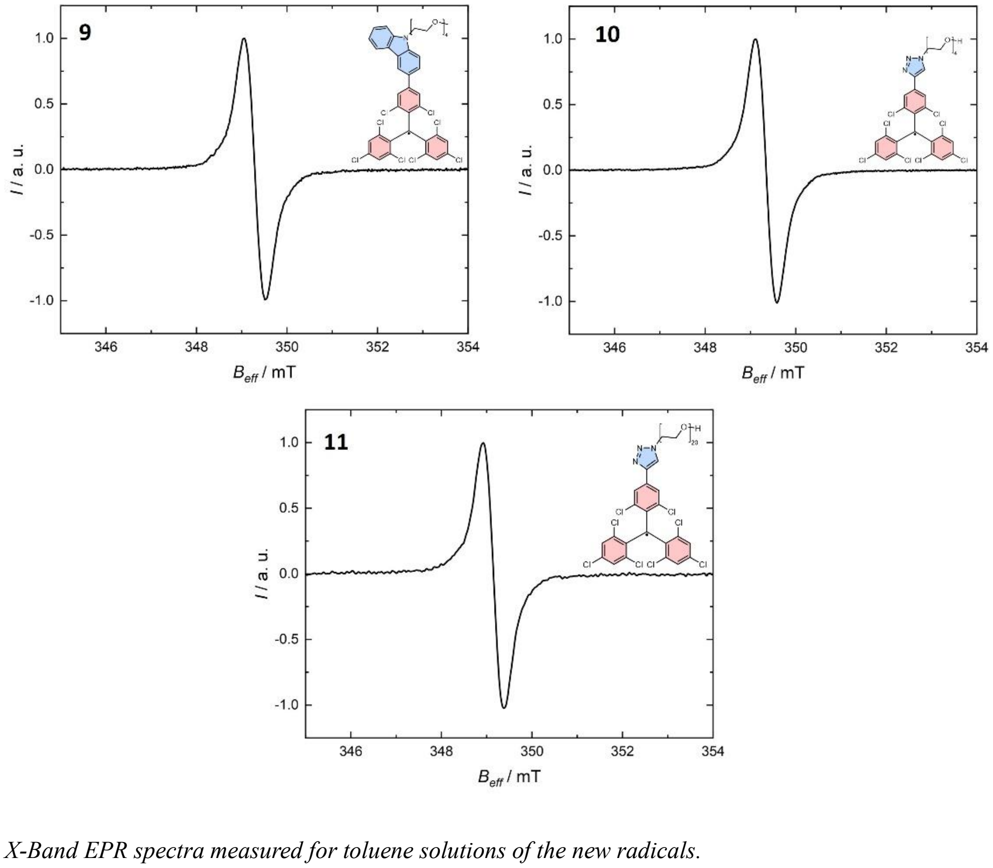

Figure S2: X-Band EPR spectra measured for toluene solutions of the new radicals.

3. Geometries obtained by DFT Calculations

Coordinates D_0 / Å

	x	y	z
C	-5.14015200	2.93054200	0.31553400
C	-4.68193100	3.73980700	-0.71166400
C	-3.62121600	3.35789900	-1.51720400
C	-3.01889900	2.13506500	-1.27885300
C	-3.43030500	1.26123000	-0.25292000
C	-4.51066200	1.71660200	0.52817000
C	-2.78405600	-0.02749900	-0.02162100
C	-3.59843600	-1.22899900	0.13717700
C	-4.64294600	-1.55804200	-0.74898100
C	-5.42959400	-2.68767300	-0.60642400
C	-5.17401400	-3.53979800	0.45596500
C	-4.16004400	-3.28094000	1.36406400
C	-3.39700100	-2.13928700	1.19367600
Cl	-5.05449000	0.79267900	1.89483000
Cl	-1.76666200	1.66312000	-2.38611200
Cl	-5.45359700	5.26651500	-0.99719200
Cl	-4.93708200	-0.58452200	-2.15722000
Cl	-2.21543600	-1.81272200	2.42420300
Cl	-6.14564400	-4.96261000	0.65523000
C	-1.33400800	-0.11480400	0.05245000
C	-0.55508800	0.77330700	0.82285200
C	0.81982600	0.69455100	0.90628700
C	1.51994900	-0.29233000	0.20503400
C	0.78227400	-1.19024900	-0.57270100
C	-0.59314200	-1.09649900	-0.63742300
Cl	-1.39688700	-2.19013100	-1.72936200
Cl	-1.32820200	1.96616900	1.82947600
C	2.98672100	-0.37828700	0.28184000
C	3.63616800	-1.62213400	0.13226000
C	5.01269700	-1.74839400	0.19819600
C	5.76149900	-0.59343100	0.41880200
C	5.13629900	0.66866400	0.56404500
C	3.75180500	0.76937600	0.49998400
N	7.12531000	-0.44168400	0.53815900
C	7.40793200	0.90242700	0.71110600
C	6.19562100	1.63089400	0.74911100
C	8.64325700	1.53238100	0.84521500
C	8.64411400	2.90890700	1.02749300
C	7.45107100	3.64465200	1.07417800
C	6.22323300	3.01286100	0.93560500
C	8.10569800	-1.47431800	0.29434800
C	8.46607700	-1.55516600	-1.17699700
O	9.43221200	-2.57298200	-1.33377600
C	9.82895000	-2.71285500	-2.68459300
H	-5.95710400	3.24493300	0.95234000
H	-3.28142500	3.98884600	-2.32836100
H	-6.21103900	-2.90621600	-1.32287100
H	-3.97769600	-3.94167000	2.20172900
H	1.34680400	1.38311000	1.55533100
H	1.28478800	-1.94439100	-1.16562900
H	3.04118400	-2.51626000	-0.01894300
H	5.48456800	-2.71811100	0.08586200
H	3.27627700	1.74035600	0.59241900
H	9.57187900	0.97392000	0.80574000
H	9.59229500	3.42593400	1.13437200
H	7.49192400	4.71913200	1.21801600
H	5.29935800	3.58176200	0.96806200
H	7.70245900	-2.42928600	0.63782500
H	8.99624000	-1.26137300	0.88986300
H	8.86865300	-0.59203800	-1.52101100
H	7.57143800	-1.78418400	-1.77313000
H	10.57026300	-3.51221300	-2.72787000
H	10.27619700	-1.78386100	-3.06072900
H	8.97386600	-2.97797000	-3.31950600

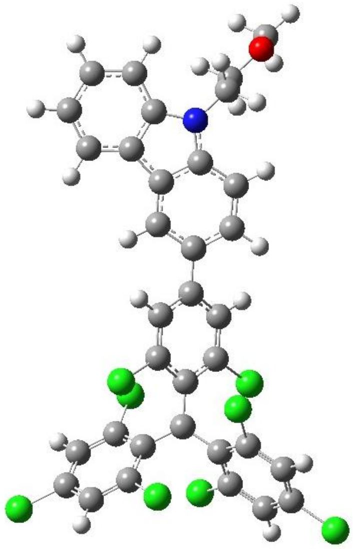

*Figure S3: Radical fragment of **9** optimized in its D_0 geometry on the PBE0-GD3(BJ)/6-311++G(d,p), SCRF (SMD, water), level of theory.*

Coordinates D_0 / Å

	x	y	z
C	2.46611100	3.49407900	-1.05262100
C	3.62210600	3.67245200	-0.30970900
C	4.09540600	2.68767200	0.54230600
C	3.38370300	1.50533400	0.64259600
C	2.20313400	1.25326000	-0.08325600
C	1.78213700	2.29744200	-0.93025000
C	1.46747000	-0.00322300	0.03066200
C	2.18347100	-1.27206800	-0.06620700
C	2.00476600	-2.31042800	0.86923200
C	2.68113000	-3.51566700	0.79929100
C	3.57818700	-3.70961800	-0.23899400
C	3.80313300	-2.73231100	-1.19506400
C	3.10667100	-1.54086500	-1.09567500
Cl	0.40164400	2.08833800	-1.96294800
Cl	3.96251700	0.35215200	1.80490300
Cl	4.49700400	5.16286400	-0.44911600
Cl	0.97231200	-2.08285400	2.24683600
Cl	3.34697500	-0.39632400	-2.37945400
Cl	4.43971200	-5.21049200	-0.34529900
C	0.02724700	0.01165600	0.24135600
C	-0.59138400	0.83611700	1.20420300
C	-1.95581600	0.86508400	1.40829000
C	-2.79389200	0.05103000	0.64522500
C	-2.22550100	-0.78559600	-0.31684500
C	-0.85823500	-0.79497500	-0.50373600
Cl	-0.25761000	-1.78830300	-1.80024400
Cl	0.37608400	1.80392300	2.27939500
C	-4.23427300	0.07816300	0.85203000
C	-5.22812400	-0.65524200	0.23592300
N	-6.36398000	-0.23382900	0.81599400
N	-6.11323700	0.70882500	1.72831500
N	-4.83135400	0.89892200	1.75941100
C	-7.72484800	-0.62200000	0.50156100
C	-8.21834000	0.10216100	-0.73618600
O	-9.54010700	-0.32902700	-0.97295700
C	-10.09770000	0.29882400	-2.11256300
H	2.11306600	4.26188400	-1.72905400
H	4.98862400	2.84254300	1.13389600
H	2.52464600	-4.27766800	1.55217300
H	4.49118400	-2.89944400	-2.01373500
H	-2.37171200	1.50546200	2.17556400
H	-2.84852400	-1.41630400	-0.93925100
H	-5.21785900	-1.41498900	-0.52948100
H	-8.33987000	-0.37377600	1.36679500
H	-7.74566500	-1.70206200	0.34957900
H	-7.57700200	-0.13595000	-1.59623000
H	-8.19086200	1.18860500	-0.57487200
H	-11.11295600	-0.08222700	-2.23051900
H	-9.51614000	0.06506700	-3.01323600
H	-10.13226100	1.38779500	-1.98268200

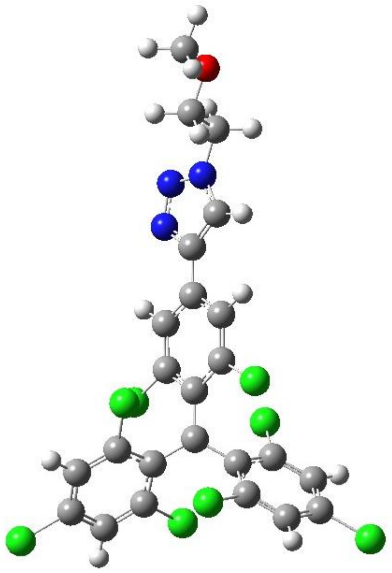

Figure S4: Radical fragment of ***10*** *and* ***11*** *optimized in its D_0 geometry on the PBE0-GD3(BJ)/6-311++G(d,p), SCRF (SMD, water), level of theory.*

4. Lippert-Mataga Plots

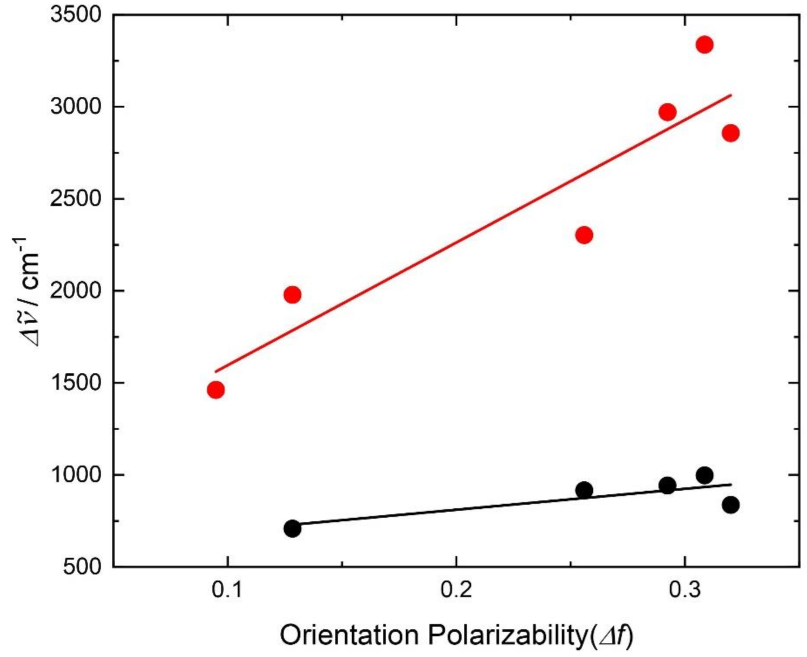

*Figure S5: Lippert–Mataga plots of radicals **9** (red) and **10** (black). The Stokes shift is plotted against the orientational polarizability Df of the solvents. While the excited state of **9** has charge transfer character as represented by the slope of the linear fit, the almost horizontal fit indicates LE behavior for **10**.*

5. DLS Measurements

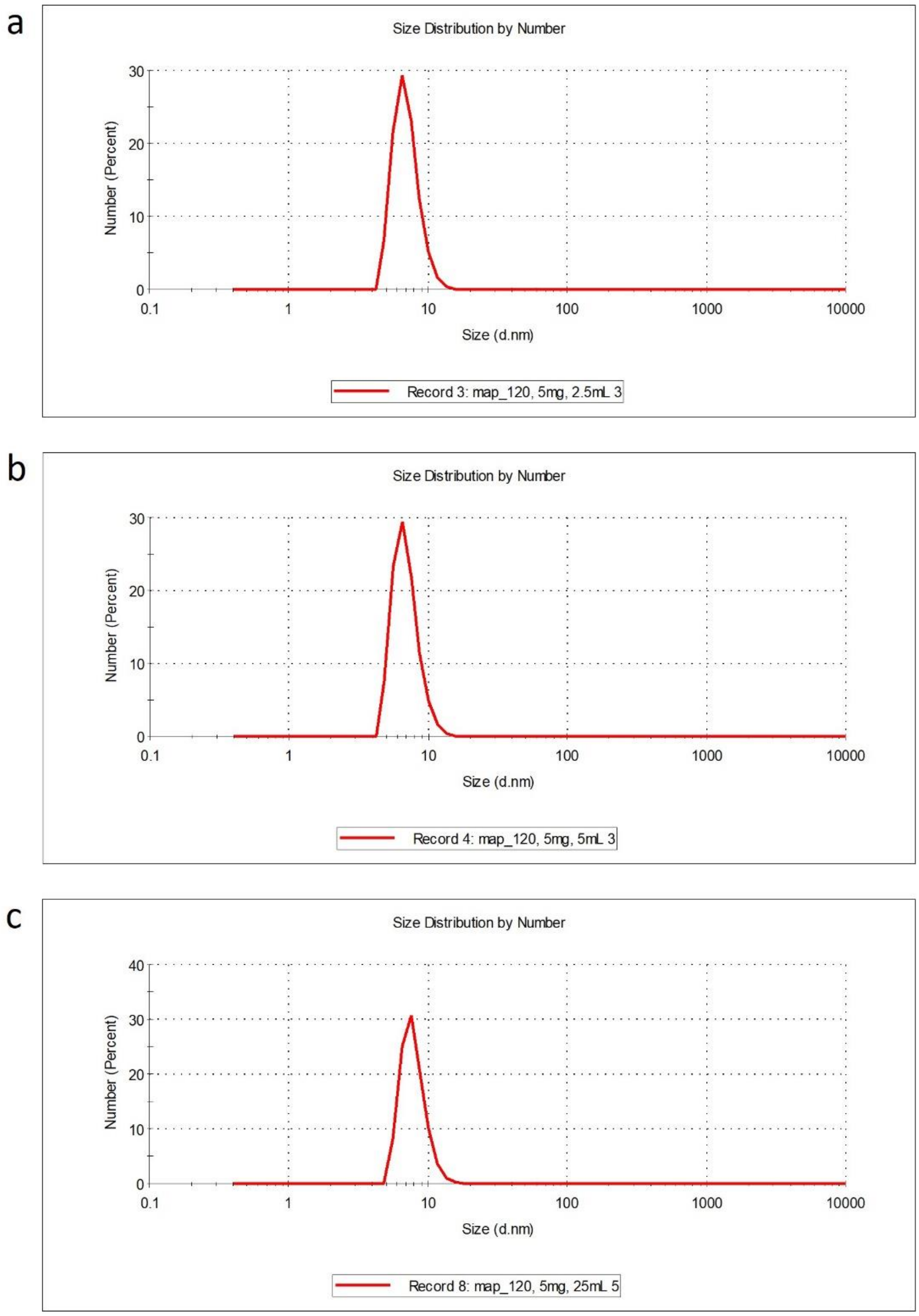

*Figure S6: DLS measurements of solutions of 5 mg **8** in 2.5 mL (a), 5 mL (b), and 25 mL (c) water.*
